# Synthesis and biological evaluation of RGD and isoDGR peptidomimetic-α-amanitin conjugates for tumor-targeting

**DOI:** 10.3762/bjoc.14.29

**Published:** 2018-02-14

**Authors:** Lizeth Bodero, Paula López Rivas, Barbara Korsak, Torsten Hechler, Andreas Pahl, Christoph Müller, Daniela Arosio, Luca Pignataro, Cesare Gennari, Umberto Piarulli

**Affiliations:** 1 Dipartimento di Scienza e Alta Tecnologia, Via Valleggio, 11, 22100, Como, Italy; 2 Dipartimento di Chimica, Università degli Studi di Milano, Via C. Golgi, 19, I-20133, Milan, Italy; 3 Heidelberg Pharma Research GmbH, Schriesheimer Strasse 101, 68526, Ladenburg, Germany; 4 CNR, Istituto di Scienze e Tecnologie Molecolare (ITSM), Via C. Golgi, 19, 20133, Milan, Italy

**Keywords:** antitumor agents, cancer, drug delivery, integrins, peptidomimetics

## Abstract

RGD-α-amanitin and isoDGR-α-amanitin conjugates were synthesized by joining integrin ligands to α-amanitin via various linkers and spacers. The conjugates were evaluated for their ability to inhibit biotinylated vitronectin binding to the purified α_V_β_3_ receptor, retaining good binding affinity, in the same nanomolar range as the free ligands. The antiproliferative activity of the conjugates was evaluated in three cell lines possessing different levels of α_V_β_3_ integrin expression: human glioblastoma U87 (α_V_β_3_+), human lung carcinoma A549 (α_V_β_3_−) and breast adenocarcinoma MDA-MB-468 (α_V_β_3_−). In the U87, in the MDA-MB-468, and partly in the A549 cancer cell lines, the cyclo[DKP-isoDGR]-α-amanitin conjugates bearing the lysosomally cleavable Val-Ala linker were found to be slightly more potent than α-amanitin. Apparently, for all these α-amanitin conjugates there is no correlation between the cytotoxicity and the expression of α_V_β_3_ integrin. To determine whether the increased cytotoxicity of the cyclo[DKP-isoDGR]-α-amanitin conjugates is governed by an integrin-mediated binding and internalization process, competition experiments were carried out in which the conjugates were tested with U87 (α_V_β_3_+, α_V_β_5_+, α_V_β_6_−, α_5_β_1_+) and MDA-MB-468 (α_V_β_3_−, α_V_β_5_+, α_V_β_6_+, α_5_β_1_−) cells in the presence of excess cilengitide, with the aim of blocking integrins on the cell surface. Using the MDA-MB-468 cell line, a fivefold increase of the IC_50_ was observed for the conjugates in the presence of excess cilengitide, which is known to strongly bind not only α_V_β_3_, but also α_V_β_5_, α_V_β_6_, and α_5_β_1_. These data indicate that in this case the cyclo[DKP-isoDGR]-α-amanitin conjugates are possibly internalized by a process mediated by integrins different from α_V_β_3_ (e.g., α_V_β_5_).

## Introduction

**Figure 1:**
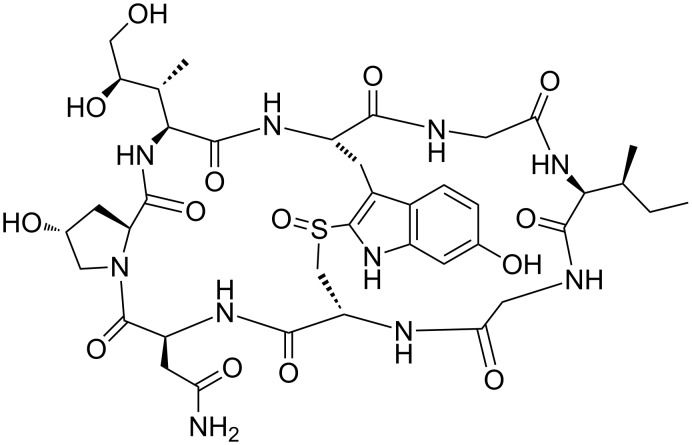
α-Amanitin.

α-Amanitin is a bicyclic octapeptide toxin belonging to the amatoxin family, found in *Amanita Phalloides* (death cap mushroom), see Figure 1 [1]. Its mechanism of action consists in the inhibition of cellular transcription by an effective blocking of RNA polymerase II, which is present in the nuclei of eukaryotic cells and is responsible for the transcription of DNA to mRNA [1–2]. Despite this strong inhibitory activity, α-amanitin exhibits only a micromolar cytotoxicity and low cellular uptake in most mammalian cells, due to its strong polarity and poor membrane permeability [2]. One notable exception are human hepatocytes, where the transporting protein OATP1B3 internalizes amatoxins resulting in high liver toxicity [2–3].

This strong toxicity in the presence of endocytosis mediators allowing cell permeation, aroused interest towards the use of α-amanitin as a payload for targeted cancer therapy. In 1981, Davis and Preston reported the synthesis of the antibody–drug conjugate (ADC) α-amanitin-anti-Thy 1.2 IgG, which was 47-fold more toxic than the unconjugated α-amanitin in the murine T lymphoma S49.1 cell line [4]. In 2012, a new ADC containing α-amanitin and a chimerized anti-EpCAM (epithelial cell-adhesion molecule) monoclonal antibody was prepared by Moldenhauer and co-workers [5]. The cytotoxicity of this conjugate was tested in EpCAM-overexpressing cancer cell lines obtaining IC_50_ values from 2.5 × 10^−10^ to 2.0 × 10^−12^ M. Promising results were also observed in mice bearing BxPc-3 pancreatic xenograft tumors, with complete tumor regression in 90% of the cases after two injections of the α-amanitin-anti-EpCAM ADC at a dose of 100 μg/kg with respect to α-amanitin. In these two examples, the internalization of the monoclonal antibody and subsequent release of the toxin leads to the enhancement of α-amanitin activity on the targeted cells.

An alternative approach to the antibody targeted therapy is represented by small molecule–drug conjugates (SMDCs), where the small molecule – usually a peptide or peptidomimetic receptor ligand – avoids the drawbacks of ADCs such as high manufacturing costs, unfavorable pharmacokinetics (low tissue diffusion and low accumulation rate) and possible elicitation of immune response [6]. By conjugation to a specific cell-membrane-receptor ligand, the toxin can be delivered at the tumor site and internalized through receptor-mediated endocytosis. In 2013, Reshetnyak and co-workers conjugated α-amanitin to pHLIP (pH low insertion peptide) via linkers of different hydrophobicities [7]. The results indicated that pHLIP could deliver α-amanitin into cells and induce cell death in 48 h by a pH-mediated direct translocation across the membrane and cleavage of the disulfide linker in the cytoplasm. In another example, Perrin and co-workers conjugated the *N*-propargylasparagine of an amanitin analog to a cycloRGD integrin ligand (cyclo[RGDfK]) using a copper-catalyzed azide–alkyne cycloaddition [8]. The conjugates were tested in the U87 glioblastoma cell line, but only a slight enhancement in toxicity over α-amanitin was observed.

**Figure 2:**
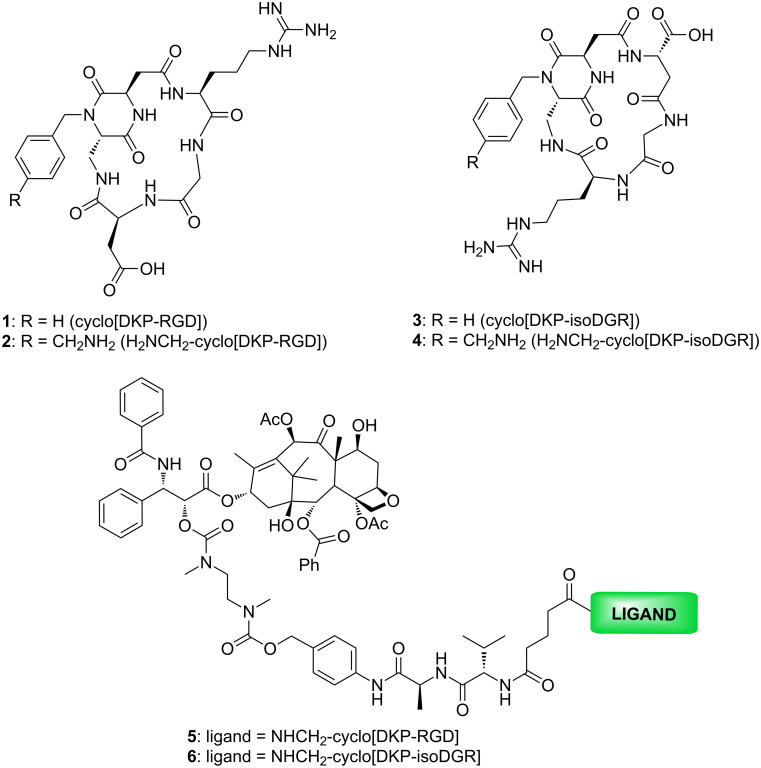
Structure of the ligands cyclo[DKP-RGD] (**1**), NH_2_CH_2_-cyclo[DKP-RGD] (**2**), cyclo[DKP-isoDGR] (**3**), NH_2_CH_2_-cyclo[DKP-isoDGR] (**4**) and the related SMDCs cyclo[DKP-RGD]-Val-Ala-PTX (**5**) and cyclo[DKP-isoDGR]-Val-Ala-PTX (**6**).

The transmembrane receptor α_V_β_3_ integrin is widely expressed on the blood vessels of several human cancers (for example, breast cancer, glioblastoma, pancreatic tumor, prostate carcinoma) but not on the vasculature of healthy tissues [9–11], and therefore constitutes a suitable therapeutic target in the field of SMDCs. Integrin α_V_β_3_ recognizes endogenous ligands by the tripeptide arginine-glycine-aspartate [12] (RGD) and also by the related sequence isoaspartate-glycine-arginine (isoDGR) [13–20]. Many synthetic peptides or peptidomimetics containing these sequences have been prepared and show low nanomolar IC_50_ values for integrin α_V_β_3_ binding [21–27]. A number of cyclic RGD and isoDGR ligands containing a bifunctional diketopiperazine (DKP) scaffold have been developed by the Gennari and Piarulli groups in the last decade [24–27]. Among them, the cyclo[DKP-RGD] **1** [25] and cyclo[DKP-isoDGR] **3** [26] (Figure 2) showed a binding affinity for the purified receptor α_V_β_3_ in the low nanomolar range and a good selectivity for this integrin in comparison with integrin α_V_β_5_ (33–34 times, see Table 1).

**Table 1:** Inhibition of biotinylated vitronectin binding to α_v_β_3_ and α_v_β_5_ receptors.

ligand	structure (name)	IC_50_ (nM)^a^ α_V_β_3_	IC_50_ (nM)^a^ α_V_β_5_

**1**	cyclo[DKP-RGD]	4.5 ± 1.1	149 ± 25
**3**	cyclo[DKP-isoDGR]	9.2 ± 1.1	312 ± 21

^a^IC_50_ values were calculated as the concentration of compound required for 50% inhibition of biotinylated vitronectin binding as estimated by GraphPad Prism software. All values are the arithmetic mean ± the standard deviation (SD) of triplicate determinations.

Furthermore, these ligands were shown to inhibit the FAK (focal adhesion kinase) and Akt (protein kinase B) signaling cascade and the tumor cell infiltration process, performing as true integrin antagonists [27].

Ligands **1** and **3** were also functionalized with an aminomethyl group (-CH_2_NH_2_) as a handle for conjugation to cytotoxic drugs (Figure 2, ligands **2** and **4**) [28–30]. Conjugates of the functionalized ligands **2** and **4** with paclitaxel (PTX) via a 2’-carbamate with a self-immolative spacer and the lysosomally cleavable Val-Ala linker [31] were synthesized (Figure 2, cyclo[DKP-RGD]-Val-Ala-PTX **5** and cyclo[DKP-isoDGR]-Val-Ala-PTX **6**). Their tumor targeting ability was assessed in vitro in antiproliferative assays comparing an α_V_β_3_ positive with an α_V_β_3_ negative cell line [29–30]. The cyclo[DKP-isoDGR]-Val-Ala-PTX conjugate **6** displayed a remarkable targeting index (TI = 9.9), especially when compared to the strictly related cyclo[DKP-RGD]-Val-Ala-PTX conjugate **5** (TI = 2.4) [30].

## Results and Discussion

**Figure 3:**
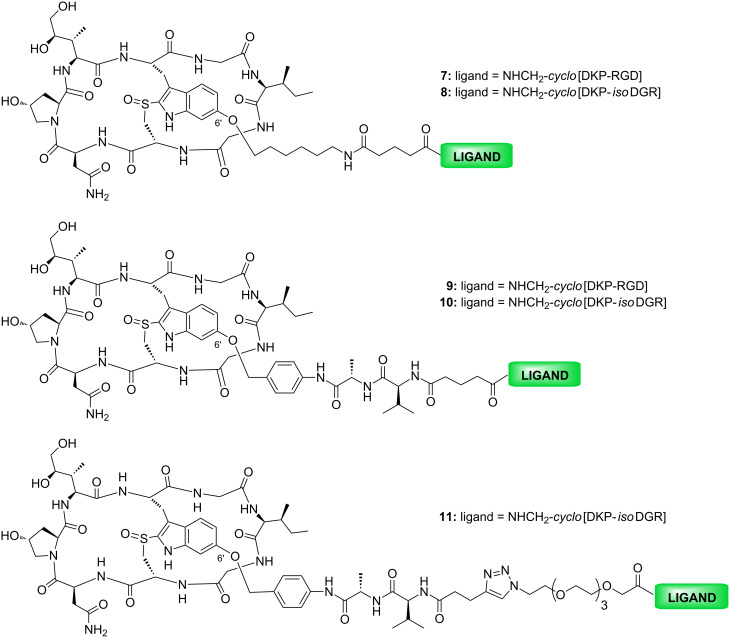
Structure of the α-amanitin conjugates: cyclo[DKP-RGD]-uncleavable-α-amanitin (**7**), cyclo[DKP-isoDGR]-uncleavable-α-amanitin (**8**), cyclo[DKP-RGD]-Val-Ala-α-amanitin (**9**), cyclo[DKP-isoDGR]-Val-Ala-α-amanitin (**10**) and cyclo[DKP-isoDGR]-PEG-4-Val-Ala-α-amanitin (**11**).

In the present paper, we report the synthesis and biological evaluation of two cyclo[DKP-RGD]-α-amanitin and three cyclo[DKP-isoDGR]-α-amanitin conjugates. In these conjugates, the integrin ligands are bound to α-amanitin via a 6’-ether with two different linkers: an “uncleavable” six carbon aliphatic chain (Figure 3, compounds **7** and **8**) and a lysosomally cleavable Val-Ala linker bound to a self-immolative spacer (Figure 3, compounds **9**, **10** and **11**). Integrin receptor competitive binding assays and cell proliferation assays with an α_V_β_3_ positive (U87) and two α_V_β_3_ negative cell lines (A549 and MDA-MB-468) were performed for all the conjugates.

### Synthesis

**Scheme 1:**
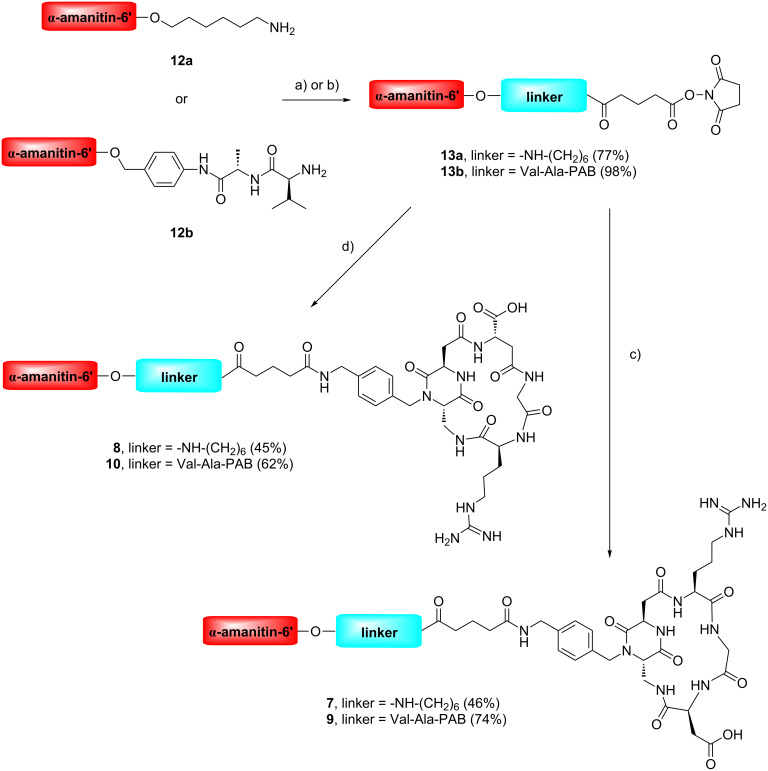
Synthesis of α-amanitin-cyclo[DKP-RGD] and α-amanitin-cyclo[DKP-isoDGR] conjugates **7**–**10**. Reagents and conditions: a) 1. glutaric anhydride, DMAP, iPr_2_NEt, DMF, overnight, 2. DIC, *N*-hydroxysuccinimide, DMF, overnight; b) di-*N*-succinimidyl glutarate, iPr_2_NEt, rt, DMF, 6 h; c) H_2_NCH_2_-cyclo[DKP-RGD] (**2**), PBS/MeCN or PBS/DMF (pH 7.5), overnight; d) H_2_NCH_2_-cyclo[DKP-isoDGR] (**4**), PBS/DMF (pH 7.5), overnight. DMAP: 4-dimethylaminopyridine; DIC: diisopropylcarbodiimide; PBS: phosphate-buffered saline; PAB: *p*-aminobenzyl.

**Scheme 2:**
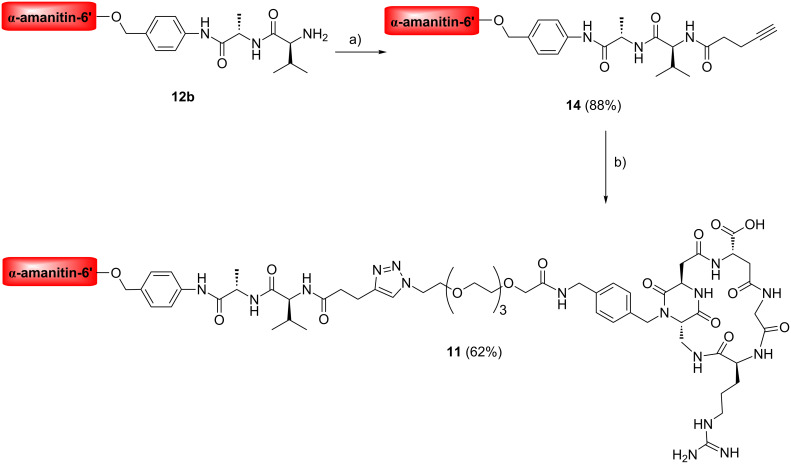
Synthesis of cyclo[DKP-isoDGR]-PEG-4-Val-Ala-α-amanitin conjugate **11**. Reagents and conditions: a) 4-pentynoic acid *N*-hydroxysuccinimidyl ester, iPr_2_NEt, DMF, overnight; b) N_3_-PEG-4-cyclo[DKP-isoDGR], sodium ascorbate, CuSO_4_·5H_2_O, DMF/water, rt, overnight.

Cyclo[DKP-RGD]-α-amanitin and cyclo[DKP-isoDGR]-α-amanitin conjugates **7**–**11** were synthesized as described in Scheme 1 and Scheme 2, by joining the functionalized ligands H_2_NCH_2_-cyclo[DKP-RGD] (**2**) [18,29] and H_2_NCH_2_-cyclo[DKP-isoDGR] (**4**) [30] to α-amanitin via a 6’-ether with various linkers and spacers. Details are reported in Supporting Information File 1.

### Integrin receptor competitive binding assays

Conjugates **7**–**11** were evaluated for their ability to inhibit biotinylated vitronectin binding to the purified α_V_β_3_ receptor. The calculated half-maximal inhibitory concentrations (IC_50_) are listed in Table 2. Screening assays were performed by incubating the immobilized integrin receptor with solutions of the cyclo[DKP-RGD]-α-amanitin and cyclo[DKP-isoDGR]-α-amanitin conjugates **7**–**11** at different concentrations (10^−12^ to 10^−5^ M) in the presence of biotinylated vitronectin (1 µg mL^−1^) and measuring bound vitronectin.

**Table 2:** Inhibition of biotinylated vitronectin binding to α_v_β_3_ receptor.

compound	structure (name)	IC_50_ (nM)^a^ α_V_β_3_

**7**	cyclo[DKP-RGD]-uncleavable-α-amanitin	11.6 ± 2.4
**8**	cyclo[DKP-isoDGR]-uncleavable-α-amanitin	6.8 ± 4.3
**9**	cyclo[DKP-RGD]-Val-Ala-α-amanitin	14.7 ± 6.6
**10**	cyclo[DKP-isoDGR]-Val-Ala-α-amanitin	6.4 ± 1.9
**11**	cyclo[DKP-isoDGR]-PEG-4-Val-Ala-α-amanitin	3.8 ± 0.3

^a^IC_50_ values were calculated as the concentration of compound required for 50% inhibition of biotinylated vitronectin binding as estimated by GraphPad Prism software. All values are the arithmetic mean ± the standard deviation (SD) of triplicate determinations.

It was found that the cyclo[DKP-RGD]-α-amanitin conjugates **7** and **9** and cyclo[DKP-isoDGR]-α-amanitin conjugates **8**, **10** and **11** retain good binding affinity for α_V_β_3_ integrin, in the same range as the free ligands (cf. Table 2 with Table 1). These results encouraged us to proceed with cell viability assays in α_V_β_3_ positive and α_V_β_3_ negative cell lines, to study the ability of the conjugates to selectively target α_V_β_3_ expressing tumor cells.

### Cell viability assays

The antiproliferative activity of the conjugates was evaluated in three cell lines expressing different levels of α_V_β_3_ integrin. U87 cells (human glioblastoma) were selected as α_V_β_3_ positive, while A549 cells (human lung carcinoma) and MDA-MB-468 (breast adenocarcinoma) [32] were used as α_V_β_3_ negative. The expression of α_V_β_3_ integrin on the cell membrane was assessed by flow cytometry (see Supporting Information File 1, Figure S1), and the results were in good agreement with the literature for U87 and A549 [33–35]. In the case of MDA-MB-468, while the presence of the β_3_ integrin subunit is still quite controversial in the literature [36–38], our FACS analysis could not detect any α_V_β_3_ expression (see Supporting Information File 1).

The cell lines were treated with different concentrations of the free drug α-amanitin and conjugates **7**–**11** for 96 hours. The cell viability was evaluated with the CellTiterGlo 2.0 assay and the calculated IC_50_ are shown in Table 3.

**Table 3:** Evaluation of anti-proliferative activity of α-amanitin and α-amanitin conjugates **7**–**11** in U-87, MDA-MB-468 and A549.

entry	structure (name)	IC_50_ (nM)^a^		
		
		U87 (α_V_β_3_+)	MDA-MB-468 (α_V_β_3_−)	A549 (α_V_β_3_−)

1	α-amanitin	347 ± 132.5^b^	185 ± 49.6^b^	518 ± 305^b^
2	cyclo[DKP-RGD]-uncleavable-α-amanitin (**7**)	2552 ± 37.6	1111 ± 228.4	n.d.^c^
3	cyclo[DKP-isoDGR]-uncleavable-α-amanitin (**8**)	3355 ± 19.1	2200 ± 96.2	n.d.^c^
4	cyclo[DKP-RGD]-Val-Ala-α-amanitin (**9**)	1446 ± 83.9	202 ± 10.3	2160 ± 23.3
5	cyclo[DKP-isoDGR]-Val-Ala-α-amanitin (**10**)	143 ± 33.8	59 ± 23.4	217 ± 98.3
6	cyclo[DKP-isoDGR]-PEG-4-Val-Ala-α-amanitin (**11**)	165 ± 4.0	66 ± 24.1	720 ± 98.1

^a^IC_50_ values were calculated as the concentration of compound required for 50% inhibition of cell viability. All cell lines were treated with different concentrations of α-amanitin and compounds **7**–**11** for 96 hours. The samples were measured in triplicate. ^b^Average values from three independent experiments. ^c^n.d.: these data could not be determined.

The cyclo[DKP-isoDGR]-α-amanitin conjugate bearing the lysosomally cleavable Val-Ala linker **10** proved slightly more potent than α-amanitin in the U87 (α_V_β_3_+) cell line, as well as in the A549 and MDA-MB-468 (α_V_β_3_−) cell lines (2.4–3.1 times, cf. entry 1 with entry 5 in Table 3). The cyclo[DKP-isoDGR]-α-amanitin conjugate bearing the lysosomally cleavable Val-Ala linker and a PEG-4 spacer **11** proved slightly more potent than α-amanitin in both the U87 (α_V_β_3_+) and MDA-MB-468 (α_V_β_3_−) cell lines (2.1–2.8 times, cf. entry 1 with entry 6 in Table 3), while in A549 cell line (α_V_β_3_−) it turned out to be less potent (1.4 times) than the free drug. Cyclo[DKP-RGD]-uncleavable-α-amanitin (**7**), cyclo[DKP-isoDGR]-uncleavable-α-amanitin (**8**) and cyclo[DKP-RGD]-Val-Ala-α-amanitin (**9**) proved less potent than α-amanitin in all cell lines (see Table 3, cf. entry 1 with entries 2–4). In general, one can conclude that the “uncleavable” compounds **7** and **8** are much less cytotoxic than free α-amanitin, while the lysosomally cleavable compounds **9**–**11** show variable results, with RGD compound **9** behaving worse than the free drug but better than the corresponding uncleavable conjugate **7**. The isoDGR motif gives generally better results, with **10** and **11** behaving much better than the corresponding uncleavable conjugate **8** and slightly better than the free drug. Apparently, for all the α-amanitin conjugates there is no direct correlation between the cytotoxicity and the expression of α_V_β_3_ integrin [39].

To determine whether the increased cytotoxicity of *cyclo*[DKP-*iso*DGR]-α-amanitin conjugates **10** and **11** is governed by an integrin-mediated binding and internalization process, competition experiments [40] were carried out in which conjugates **10** and **11** were tested on U87 (α_V_β_3_+, α_V_β_5_+, α_V_β_6_−, α_5_β_1_+) [34–36] and on MDA-MB-468 (α_V_β_3_−, α_V_β_5_+, α_V_β_6_+, α_5_β_1_−) [34–36] cells in the presence of 50-fold excess of cilengitide [23], with the aim of blocking integrins on the cell surface (Table 4, see also Supporting Information File 1, Biological assays). Using the U87 cell line, a modest IC_50_ increase of conjugate **11** from 91 nM (without cilengitide) to 143 nM (with excess cilengitide) was observed (Table 4, entry 2). Using the MDA-MB-468 cell line, a more pronounced IC_50_ increase was observed for both conjugates **10** (from 47 nM without cilengitide to 259 nM with excess cilengitide; Table 4, entry 1) and **11** (from 65 nM without cilengitide to 340 nM with excess cilengitide; Table 4, entry 2). From these results, no correlation emerges between the expression of integrin α_V_β_3_ and cytotoxicity. However, it should be noted that these cell lines overexpress other integrins (α_5_β_1_ in U87, α_V_β_6_ in MDA-MB-468, and α_V_β_5_ in both) [34–36]. Thus, the increase of the IC_50_ of conjugates **10** and **11** (up to 5.5 times) may be possibly due to the block of other integrins with excess cilengitide, which is known to efficiently bind not only α_V_β_3_ (IC_50_ = 0.6 nM) [23], but also α_V_β_5_ (IC_50_ = 8.4 nM) [23], α_V_β_6_ (IC_50_ = 82.8 nM) [41], and α_5_β_1_ (IC_50_ = 14.9 nM) [23].

**Table 4:** Competition experiments of conjugates **10** and **11** in the presence of a 50-fold excess of cilengitide in U-87 and MDA-MB-468.

entry	compound	IC_50_ (nM)^a^	

		U87 (α_V_β_3_+, α_V_β_5_+, α_V_β_6_−, α_5_β_1_+)	MDA-MB-468 (α_V_β_3_−, α_V_β_5_+, α_V_β_6_+, α_5_β_1_−)

1	**10**	107 ± 26.8	47 ± 21.1
	**10** + 50-fold excess of cilengitide	106 ± 11.6	259 ± 55.2

2	**11**	91 ± 30.6	65 ± 17.6
	**11** + 50-fold excess of cilengitide	143 ± 59.3	340 ± 210.3

^a^IC_50_ values were calculated as the concentration of compound required for 50% inhibition of cell viability. Both cell lines were treated with different concentrations of compounds **10** and **11** in the presence of a 50-fold excess of cilengitide during 96 hours. The samples were measured in triplicate*.*

## Conclusion

In this paper, two cyclo[DKP-RGD]-α-amanitin and three cyclo[DKP-isoDGR]-α-amanitin conjugates were prepared in good yields following a straightforward synthetic route. Conjugates **7**–**11** retain good binding affinities for the purified α_V_β_3_ receptor, in the same range as the respective free ligands. Cell proliferation assays were performed with three cell lines possessing different levels of integrin expression: human glioblastoma U87 (α_V_β_3_+), human lung carcinoma A549 (α_V_β_3_−) and breast adenocarcinoma MDA-MB-468 (α_V_β_3_−). With all these cell lines the cyclo[DKP-isoDGR]-α-amanitin conjugate **10** proved slightly more potent than α-amanitin, whereas conjugate **11** showed enhanced potency compared to the free drug only on U87 and MDA-MB-468 cells.

Apparently, for all these α-amanitin conjugates there is no correlation between the cytotoxicity and the expression of α_V_β_3_ integrin. To determine whether the slightly increased cytotoxicity of cyclo[DKP-isoDGR]-α-amanitin conjugates **10** and **11** is governed by an integrin-mediated binding and internalization process, competition experiments were carried out in which conjugates **10** and **11** were tested with U87 (α_V_β_3_+, α_V_β_5_+, α_V_β_6_−, α_5_β_1_+) and MDA-MB-468 (α_V_β_3_−, α_V_β_5_+, α_V_β_6_+, α_5_β_1_−) cells in the presence of 50-fold excess of cilengitide, with the aim of blocking integrins on the cell surface. Using the U87 cell line, a modest increase of the conjugate **11** IC_50_ was observed in the presence of cilengitide. Employing the MDA-MB-468 cell line, a more pronounced increase of IC_50_ was observed for both conjugates **10** and **11** in the presence of cilengitide. Therefore, it appears that blocking integrins with excess cilengitide, which is known to strongly bind not only α_V_β_3_, but also α_V_β_5_, α_V_β_6_, and α_5_β_1_, results in an increase (up to 5.5 times) of the IC_50_ of conjugates **10** and **11** with the MDA-MB-468 cell line. These data suggest that cyclo[DKP-isoDGR]-α-amanitin conjugates **10** and **11** are possibly internalized by a process mediated by integrins different from α_V_β_3_ (e.g., α_V_β_5_), though the exact nature of this involvement is not clearly defined [42].

Finally, the IC_50_ values of the integrin ligand-α-amanitin conjugates are much worse (cytotoxicity increased three times compared to α-amanitin) than those exhibited by antibody α-amanitin conjugates for which the increase of cytotoxicity is 100–100000 times [5]. Therefore, despite the remarkable progresses that have been realized in recent years, integrin targeting SMDCs are still far from the clinic.

## Experimental

Functionalized ligands H_2_NCH_2_-cyclo[DKP-RGD] (**2**) and H_2_NCH_2_-cyclo[DKP-isoDGR] (**4**) were prepared according to the literature [28–29]. 6’-Functionalized α-amanitin derivatives **12a** and **12b** were prepared according to references [43–44].

## Supporting Information

File 1 Experimental details, characterization data and copies of spectra.
