# Supplementary material for: Synthesis and biological evaluation of RGD and isoDGR peptidomimetic-α-amanitin conjugates for tumor-targeting
Source: Beilstein J Org Chem. 2018 Feb 14;14:407–15. doi: 10.3762/bjoc.14.29 (PMC5827777; doi:10.3762/bjoc.14.29)

Supporting Information

for

Synthesis and biological evaluation of RGD and isoDGR peptidomimetic-α-amanitin conjugates for tumor-targeting

Lizeth Bodero[‡1], Paula López Rivas[‡2], Barbara Korsak[3], Torsten Hechler[3], Andreas Pahl[3], Christoph Müller[3], Daniela Arosio[4], Luca Pignataro[2], Cesare Gennari*[2] and Umberto Piarulli*[1]

Address: [1]Dipartimento di Scienza e Alta Tecnologia, Via Valleggio, 11, 22100, Como (Italy), [2]Dipartimento di Chimica, Università degli Studi di Milano, Via C. Golgi, 19, I-20133, Milan (Italy), [3]Heidelberg Pharma Research GmbH, Schriesheimer Strasse 101, 68526, Ladenburg (Germany) and [4]CNR, Istituto di Scienze e Tecnologie Molecolare (ITSM), Via C. Golgi, 19,20133, Milan (Italy).

Email: Cesare Gennari* - cesare.gennari@unimi.it; Umberto Piarulli* - umberto.piarulli@uninsubria.it

*Corresponding author

[‡]Equal contributors

Experimental details, characterization data and copies of spectra

Table of contents

Material and methods **S2**

Biological assays **S3**

Solid phase receptor binding assays S3
Determination of integrin $\alpha_V\beta_3$ expression by flow cytometry S4
Cell culture S4
Cell therapy and viability assay S5
Competition experiment of compounds **10** and **11** S5

Synthesis of cyclo[DKP-RGD]-α-amanitin conjugates 7 and 9 **S6**

Synthesis of cyclo[DKP-RGD]-uncleavable-α-amanitin (**7**) S6
Synthesis of cyclo[DKP-RGD]-Val-Ala-α-amanitin (**9**) S9

Synthesis of cyclo[DKP-isoDGR]-α-amanitin conjugates 8, 10 and 11 **S11**

Synthesis of cyclo[DKP-isoDGR]-uncleavable-α-amanitin (**8**) S11
Synthesis of cyclo[DKP-isoDGR]-Val-Ala-α-amanitin (**10**) S13
Synthesis of cyclo[DKP-isoDGR]-PEG-4-Val-Ala-α-amanitin (**11**) S15

HPLC traces of the final compounds **S18**

ESI–MS, MALDI–TOF–MS and HRMS Spectra **S20**

^{1}H NMR and ^{13}C NMR spectra **S25**

Material and methods

All manipulations requiring anhydrous conditions were carried out in flame-dried glassware, with magnetic stirring and under a nitrogen atmosphere. All commercially available reagents were used as received. Anhydrous solvents were purchased from commercial sources and withdrawn from the container by syringe, under a slight positive pressure of nitrogen. The reactions were monitored by analytical thin-layer chromatography (TLC) using Macherey-Nagel 0.20 mm silica gel 60 with fluorescent indicator pre-coated polyester sheets (40 × 80 mm). Visualization was accomplished by irradiation with a UV lamp and/or staining with cerium/molybdate reagent, ninhydrin or cinnamaldehyde. Automated chromatography was performed with Teledyne Isco CombiFlash Rf 150. Proton NMR spectra were recorded on a spectrometer operating at 400.16 MHz. Proton chemical shifts are reported in ppm (δ) with the solvent reference relative to tetramethylsilane (TMS) employed as the internal standard (D_2O δ = 4.79 ppm). The following abbreviations are used to describe spin multiplicity: s = singlet, d = doublet, t = triplet, q = quartet, m = multiplet, bs = broad signal, dd = doublet of doublet, ddd = doublet of doublet of doublet, ddt = doublet of doublet of triplet, td= triplet of doublet. Carbon NMR spectra were recorded on a spectrometer operating at 100.63 MHz, with complete proton decoupling. Carbon chemical shifts are reported in ppm (δ) relative to TMS with the respective solvent resonance as the internal standard.

ESI–MS spectra were recorded on an ion trap mass spectrometer Finnigan LCQ Advantage or Micro Waters Q-Tof (ESI source) and on a Thermo Fisher linear ion trap LTQ XL mass spectrometer. The MALDI–TOF–MS spectra were recorded on a Bruker Microflex™ LT instrument, supporting the sample on α-cyano-4-hydroxycinnamic acid (HCCA) and sinapinic acid (SA) matrices. The peptide calibration standard (300–3000 Da range), which consisted of angiotensin II, angiotensin I, substance P, bombesin; ACTH clip 1-17, ACTH clip 18-39, somatostatin 28, was purchased from Bruker Daltonics® and used to calibrate the MALDI-TOF-MS instrument. The sample was mixed in equal volumes with the matrix solution: a small amount (1 μL) of this mixture was spotted on the target surface. The target matrix was dried at room temperature and then analyzed.

High-resolution mass spectra (HRMS) were performed with a Fourier Transform Ion Cyclotron Resonance (FT-ICR) Mass Spectrometer APEX II & Xmass software (Bruker Daltonics) – 4.7 T Magnet (Magnex) equipped with ESI source, available at CIGA (Centro Interdipartimentale Grandi Apparecchiature) c/o Università degli Studi di Milano.

HPLC purifications and HPLC traces of final products were performed on Dionex Ultimate 3000 equipped with Dionex RS Variable Wavelenght Detector (column: Atlantis Prep T3 OBD^{TM} 5 µm 19 × 100 mm; flow 15 mL/min unless stated otherwise). The crude reaction mixture was dissolved in H_2O or, if the compound was insoluble in water, adding first DMF, then diluting slowly with H_2O until reaching a 1:1 DMF/H_2O ratio (an ultrasonic bath was used

to assist the dissolution). The solution obtained was filtered (polypropylene, 0.45 μm, 13 mm ø, PK/100) and injected in the HPLC, affording purified products. Purity analyses were carried on a Dionex Ultimate 3000 instrument equipped with a Dionex RS Variable Wavelenght detector (column: Atlantis® Prep T3 OBDTM 5 μm 19 × 100 mm). The analyte (1 mg) was dissolved in 1 mL of H_2O and was injected using the same gradient used in the purification step. The analysis of the integrals and the relative percentage of purity were performed with the software Chromeleon 6.80 SR11 Build 3161. Also, preparative HPLC LaPrep∑ equipped with autosampler AS3950 and a Phenomenex Luna C-18(2) column, 10 µm, 250 × 21.2 mm, with precolumn at 30 mL/min flow rate and analytical HPLC performed on Hitachi Chromaster (column oven Chromaster 5310, pump Chromaster 5110, autosampler Chromaster 5210, DAD Chromaster 5430) equipped with a Phenomenex Luna C-18(2) column, 10 µm, 250 × 4.6 mm, with precolumn at 1.4 mL/min flow rate were used. Freeze-drying: The product was dissolved in water and frozen with dry ice. The freeze-drying was carried out at least for 48 h at −50 °C using the instrument 5Pascal Lio5P DGT.

Biological assays

Solid phase receptor binding assays

Recombinant human integrin $\alpha_V\beta_3$ (R&D Systems, Minneapolis, MN, USA) was diluted to 0.5 μg/mL in coating buffer containing 20 mM Tris-HCl (pH 7.4), 150 mM NaCl, 1 mM $MnCl_2$, 2 mM $CaCl_2$, and 1 mM $MgCl_2$. 100 Microliters of the diluted receptor (100 μL/well) were added to 96-well microtiter plates (Nunc MaxiSorp) and incubated overnight at 4 °C. The plates were treated for additional 2 h at room temperature with blocking solution (coating buffer plus 1% bovine serum albumin) to block nonspecific binding, and washed 2 times with the same solution. Different concentrations (10^{-5}–10^{-12} M) of the test compounds in the presence of 1 μg/mL biotinylated vitronectin were added to the plates, which were shaken for 3 h at room temperature. Vitronectin,(Molecular Innovations, Novi, MI, USA) was biotinylated using an EZ-Link Sulfo-NHS-Biotinylation kit (Pierce, Rockford, IL, USA). The plates were washed 3 times, and incubated with shaking for 1 h, at room temperature, with streptavidin-biotinylated peroxidase complex (Amersham Biosciences, Uppsala, Sweden). The plates were again washed 3 times with blocking solution, and 100 μL/well of Substrate Reagent Solution (R&D Systems, Minneapolis, MN, USA) were added before shaking in the dark for 30 min and stopping the reaction with the addition of 50 μL/well 2 N H_2SO_4. Absorbance at 415 nm was read in a SynergyTM HT Multi-Detection Microplate Reader (BioTek Instruments, Inc.). Each data point represents the average of triplicate wells; data analysis was carried out by nonlinear regression analysis with GraphPad Prism software. Each experiment was repeated in triplicate.

Determination of integrin $\alpha_V\beta_3$ expression by flow cytometry

The expression of integrin $\alpha_V\beta_3$ in U87-MG, A549 and MDA-MB 468 cells was determined by flow cytometry on a FACSCalibur device (Becton Dickinson). Before staining, cells were fixed with fixation solution (0.5% PFA in PBS). 5×10^5 cells per sample were stained in staining medium (PBS, 25 mM HEPES, 3% FCS, 0.02% Na-azide) with an anti-human integrin $\alpha_V\beta_3$ antibody conjugated to Alexa Fluor 488 (R&D Systems) or isotype control conjugated to Alexa Fluor 488 (Thermo Fischer) at a concentration of 4 µg/mL for 45 min at room temperature. The cells were washed with PBS and the mean fluorescence intensity was measured for 10.000 gated events. The data were analyzed using flow cytometry and associated software (BD Biosciences) (Figure S1).

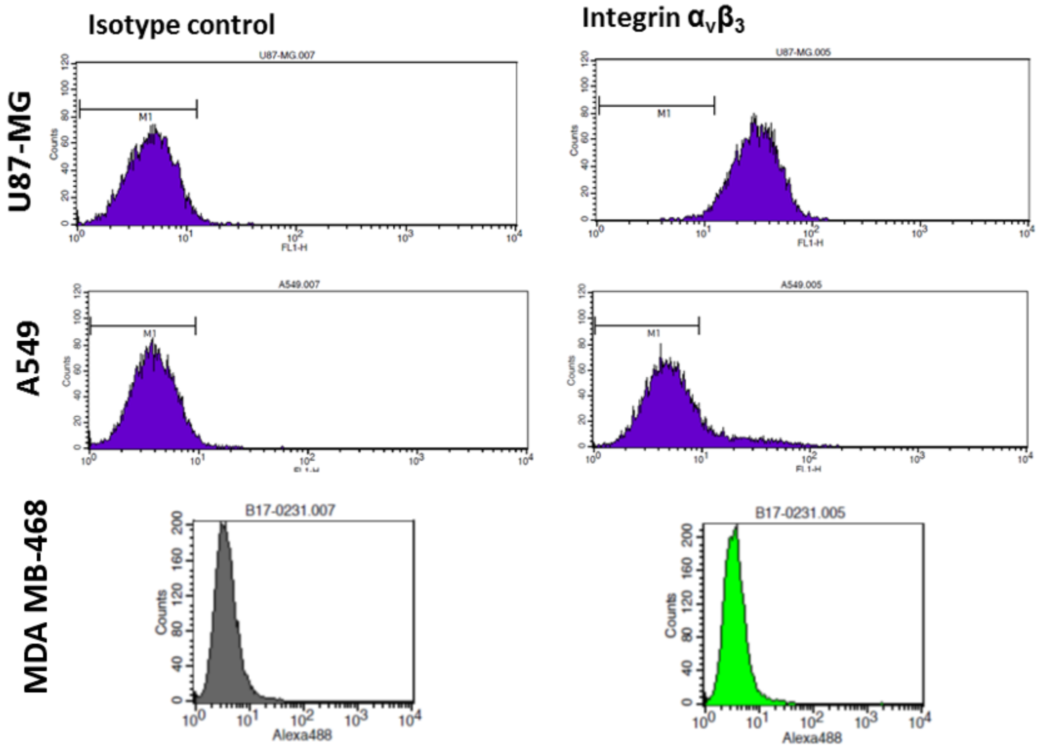

Figure S1. Flow cytometry analysis of integrin $\alpha_V\beta_3$ expression in cancer cell lines. U87-MG: integrin $\alpha_V\beta_3$ overexpressed; A549 and MDA-MB 468: integrin $\alpha_V\beta_3$ negative.

Cell culture

All cell culture reagents were purchased at PAN-Biotech GmbH unless otherwise stated. Cell lines were obtained from CLS (U87-MG, MDA-MB 468 and A549). Cell lines were authenticated using Multiplex Cell Authentication by Multiplexion (Heidelberg, Germany) as

described recently.[S1] The SNP profiles matched known profiles or were unique. The purity of the cell lines was validated using the Multiplex cell Contamination Test by Multiplexion (Heidelberg, Germany) as described recently.[S2] No mycoplasma, SMRV or interspecies contamination was detected. U87-MG, MDA-MB 468 and A549 cells were cultivated continuously for not more than 3 months in MEM Eagle´s, DMEM or Ham´s F12 medium, respectively, supplemented with 10% heat inactivated fetus calf serum, L-glutamine and antibiotics. Cell lines were maintained at 37 ºC and 5% CO_2 in a high humidity atmosphere.

Cell therapy and viability assay

Cell viability assays were performed in U87-MG, A549 and MDA-MB 468 cell lines according to the following procedure: 2×10^3 cells/well were plated in 96-well black clear bottom plates (Perkin Elmer) and incubated overnight. 1:5 serial dilutions of compounds **7–11** were prepared in cell culture media. The compounds were added to the cell culture and incubated for additional 96 h. Starting concentration of compounds **7–11** in the wells was 1×10^{-5} M and cell viability was determined with the CellTiterGlo 2.0 assay (Promega) in accordance to manufacturer's instructions. The cell viability was calculated in relation to the non-treated controls for each cell line. All samples were measured in triplicate. Data analysis was carried out using software GraphPad Prism (GraphPad Software Inc., La Jolla, CA, USA).

Competition experiment of compounds **10** and **11**

The competition experiments were performed in U87-MG and MDA-MB 468 cell lines according to the following procedure: 2×10^3 cells/well were plated in 96-well black clear bottom plates (Perkin Elmer) and incubated overnight. A solution containing 1×10^{-4} M of the conjugates **10** or **11** and 5×10^{-3} M of cilengitide (50-fold excess of ligand in comparison to the conjugate) was prepared in the growth medium and 1:5 serial dilutions were prepared in cell culture media. The compounds were added to cell culture and incubated for 96 h. The starting concentration of the compounds in the wells was 0.01 mM whereas that of cilengitide was 0.5 mM. The cell viability was determined with the CellTiterGlo 2.0 assay (Promega) in accordance to manufacturer's instructions and the viability was calculated in relation to the non-treated controls for each cell line. All samples were measured in triplicate. Data analysis was carried out using software GraphPad Prism (GraphPad Software Inc., La Jolla, CA, USA).

[S1] F. Castro, W. G. Dirks, S. Fähnrich, A. Hotz-Wagenblatt, M. Pawlita, M. Schmitt, *Int. J. Cancer* **2013**, *132*, 308–314.
[S2] M. Schmitt, M. Pawlita, *Nucleic Acids Res.* **2009**, *37*, e119.

Synthesis of cyclo[DKP-RGD]-α-amanitin conjugates 7 and 9

Synthesis of cyclo[DKP-RGD]-uncleavable-α-amanitin (7)

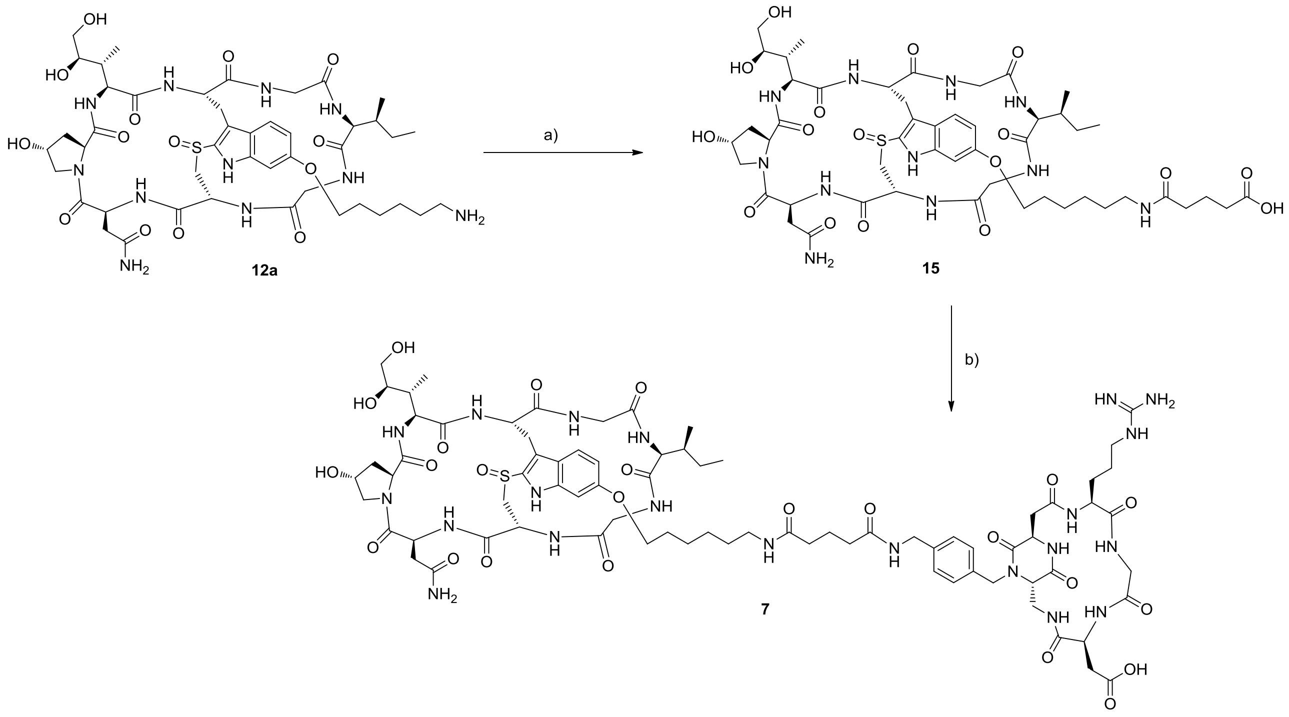

Scheme S1. Synthesis of cyclo[DKP-RGD]-uncleavable-α-amanitin (**7**). Reagents and conditions: a) glutaric anhydride, DMAP, iPr_2NEt, DMF, overnight, yield.: 77%; b) 1. DIC, *N*-hydroxysuccinimide, DMF, overnight, 2. NH_2CH_2-cyclo[DKP-RGD] (**2**), PBS/MeCN (pH 7.5), overnight, yield.: 46%.

Hemiglutarate-aminohexyl-α-amanitin (**15**)

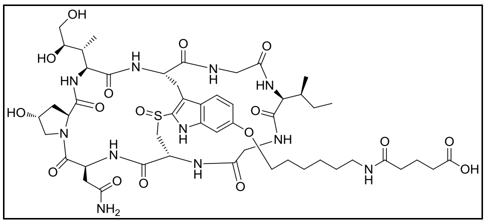

Compound **12a** (5 mg, 4.4 µmol, 1 equiv) was dissolved in 10 µL of DMF and cooled at 0 ºC under nitrogen conditions. Glutaric anhydride (1.26 mg, 11 µmol, 2.5 equiv), DMAP (0.13 mg, 1.1 µmol, 0.25 equiv) and iPr_2NEt (2.87 µL, 16.5 µmol, 3.75 equiv) were added as stock solutions in DMF and the reaction was stirred at 0 ºC for a few minutes. The mixture was then warmed to room temperature and stirred overnight under nitrogen atmosphere. The solvent was evaporated under high vacuum and the crude was purified by semipreparative HPLC [Waters

Atlantis 21 mm × 10 cm column; gradient: 100% (H_2O + 0.1 % CF_3COOH)/0% (CH_3CN + 0.1% CF_3COOH) to 55% (H_2O + 0.1 % CF_3COOH)/45% (CH_3CN + 0.1% CF_3COOH) in 10 minutes; t_R: (product): 8.8 min]. The purified compound was then freeze-dried to affford compound **15** as a white solid (3.85 mg, 77% yield).

^{1}H NMR (400 MHz, D_2O) δ 8.70 (d, *J* = 9.8 Hz, 1H), 8.25 (d, *J* = 7.1 Hz, 1H), 7.77 (d, *J* = 8.9 Hz, 1H), 7.10 (d, *J* = 2.0 Hz, 1H), 6.96 (dd, *J* = 8.9, 2.1 Hz, 1H), 5.26 (td, *J* = 11.4, 8.1 Hz, 1H), 5.11 (dd, *J* = 12.8, 4.7 Hz, 1H), 5.00 (s, 1H), 4.74 (s, 1H), 4.67 – 4.61 (m, 2H), 4.26 – 4.09 (m, 5H), 3.88 (d, *J* = 13.7 Hz, 1H), 3.82 (d, *J* =18.8 Hz, 2H), 3.77 – 3.71 (m, 5H), 3.67 – 3.52 (m, 3H), 3.30 – 3.20 (m, 4H), 2.94 (dd, *J* = 14.6, 11.8 Hz, 1H), 2.58 – 2.50 (m, 1H), 2.49 – 2.41 (m, 1H), 2.34 – 2.28 (m, 2H), 2.27 – 2.14 (m, 3H), 1.89 – 1.79 (m, 4H), 1.78 – 1.70 (m, 1H), 1.61 – 1.48 (m, 5H), 1.47 – 1.38 (m, 2H), 1.29 – 1.19 (m, 1H), 1.03 (d, *J* = 7.0 Hz, 3H), 0.92 – 0.86 (m, 6H); ^{13}C NMR (101 MHz, D_2O) δ 174.86, 174.17, 173.79, 173.27, 171.88, 171.65, 170.91, 170.17, 168.65, 168.53, 157.00, 139.14, 127.40, 122.47, 121.38, 114.35, 111.87, 96.07, 72.73, 69.84, 68.81, 63.36, 62.08, 59.91, 56.63, 55.73, 52.89, 51.60, 50.64, 42.58, 41.68, 39.12, 37.87, 37.21, 34.85, 33.30, 32.69, 28.06, 25.44, 25.33, 24.76, 20.76, 14.12, 12.96, 9.80; MS (ESI+) *m/z* calcd. for $[C_{50}H_{74}N_{11}O_{17}S]^+$: 1132.50 $[M + H]^+$; found: 1132.87; *m/z* calcd. for $[C_{50}H_{73}N_{11}NaO_{17}S]^+$: 1154.48 $[M + Na]^+$; found: 1154.86; *m/z* calcd. for $[C_{50}H_{75}N_{11}O_{17}S]^{2+}$: 566.75 $[M + 2H]^{2+}$; found: 566.95; *m/z* calcd. for $[C_{50}H_{74}N_{11}NaO_{17}S]^{2+}$: 578.25 $[M + 1H + Na]^{2+}$; found: 577.94.

Cyclo[DKP-RGD]-uncleavable-α-amanitin (**7**)

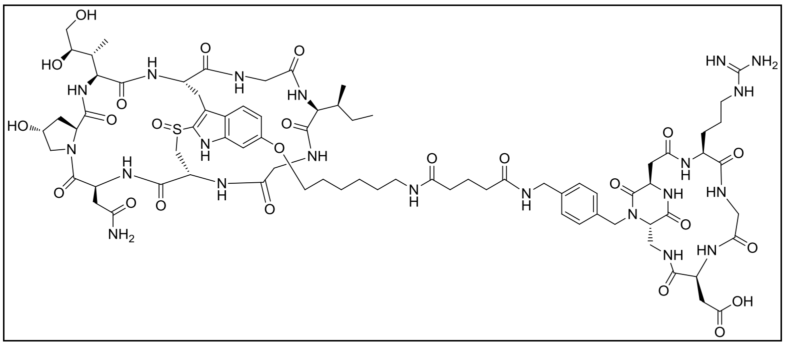

Compound **15** (7.58 mg, 6.7 μmol, 1 equiv) was dissolved in 175 μL of DMF and cooled at 0 ºC under a nitrogen atmosphere. *N*-hydroxysuccinimide (1.15 mg, 10 μmol, 1.5 equiv) and DIC (1.55 μL, 10 μmol, 1.5 equiv) were added as stock solutions in DMF and the reaction mixture was stirred at 0 ºC for a few minutes. The reaction was allowed to reach room temperature and stirred overnight under a nitrogen atmosphere. Volatiles were then removed in vacuo to give a solid, which was redissolved in acetonitrile (176 μL) and cooled to 0 ºC. A solution of NH_2CH_2-

cyclo[DKP-RGD] (**2**) (2.88 mg, 3.35 μmol, 0.5 equiv) in phosphate buffer (211 μL, pH 7.5) was then added to the acetonitrile solution, and the pH value was adjust to 7.3–7.6 with NaOH (0.2 M). The resulting solution was warmed to room temperature and stirred overnight. During the first hours, the pH was kept between 7.3–7.6 with NaOH (0.2 M), until a stable value was observed. The solution was concentrated, and the crude residue was purified by semipreparative HPLC [Waters Atlantis 21 mm × 10 cm column; gradient: 100% (H_2O + 0.1% CF_3COOH)/0% (CH_3CN + 0.1% CF_3COOH) to 50% (H_2O + 0.1% CF_3COOH)/ 50% (CH_3CN + 0.1% CF_3COOH) in 9 minutes; t_R: (product): 8 min]. The purified compound was then freeze-dried to afford compound **7** as a white solid (2.87 mg, 46% yield).

^{1}H NMR (400 MHz, D_2O) δ 8.70 (s, 1H), 8.27 (d, *J* = 8.2 Hz, 1H), 7.68 (d, *J* = 8.9 Hz, 1H), 7.26 (m, 4H), 7.01 (d, *J* = 2.1 Hz, 1H), 6.88 (dd, *J* = 8.8, 2.1 Hz, 1H), 5.30 – 5.19 (m, 1H), 5.11 (dd, *J* = 9.5, 4.7 Hz, 1H), 5.03 – 4.97 (m, 2H), 4.88 (t, *J* = 7.1 Hz, 1H), 4.75 (s, 1H), 4.68 – 4.61 (m, 2H), 4.55 (dd, *J* = 7.9, 5.6 Hz, 1H), 4.39 – 4.04 (m, 11H), 4.00 – 3.79 (m, 3H), 3.79 – 3.48 (m, 10H), 3.31 – 3.14 (m, 6H), 2.99 – 2.74 (m, 2H), 2.66 – 2.43 (m, 1H), 2.34 – 2.12 (m, 4H), 2.05 – 1.87 (m, 2H), 1.85 – 1.70 (m, 3H), 1.71 – 1.60 (m, 1H), 1.57 – 1.44 (m, 4H), 1.43 – 1.33 (m, 2H), 1.26 – 1.20 (m, 2H), 1.15 (d, *J* = 6.5 Hz, 8H), 1.04 (d, *J* = 7.0 Hz, 3H), 0.95 – 0.85 (m, 6H). MS (MALDI-TOF): *m/z* calcd. for $[C_{77}H_{110}N_{21}O_{24}S]^+$: 1745.89 $[M + H]^+$; found: 1746.5 (HCCA matrix), 1747.9 (SA matrix).

Synthesis of cyclo[DKP-RGD]-Val-Ala-α-amanitin (**9**)

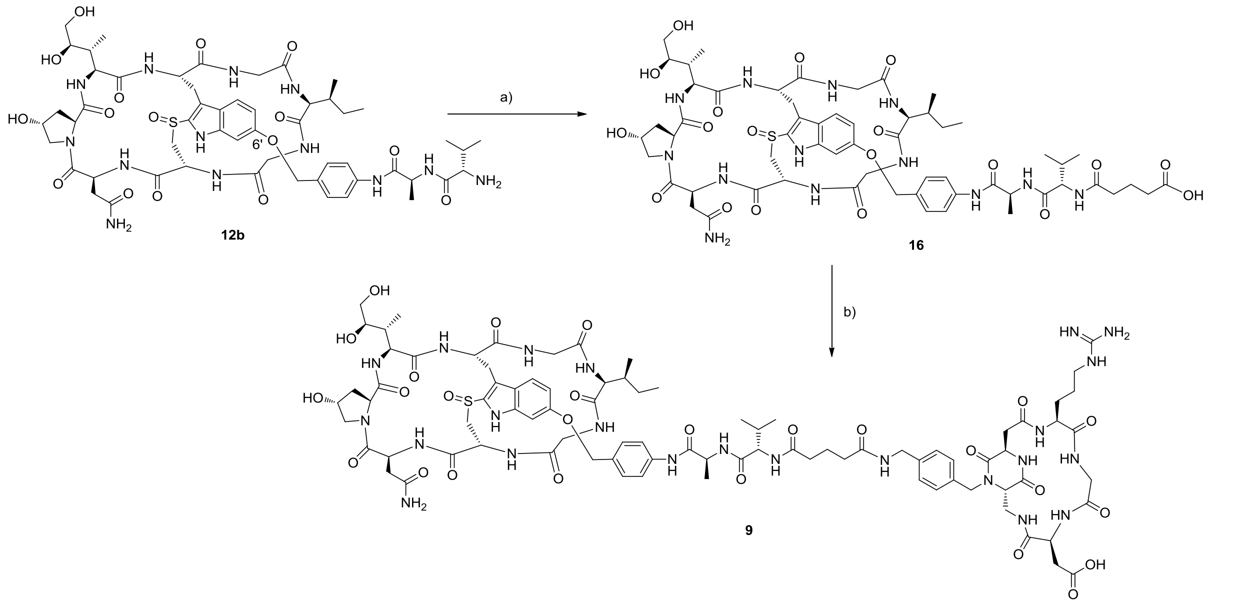

Scheme S2. Synthesis of cyclo[DKP-RGD]-Val-Ala-α-amanitin (**9**). Reagents and conditions: a) glutaric anhydride, DMAP, iPr_2NEt, DMF, overnight, yield.: 98%; b) 1. DIC, *N*-hydroxysuccinimide, DMF, overnight, 2. NH_2CH_2-cyclo[DKP-RGD] (**2**), PBS (pH 7.5), overnight, yield: 74%.

Hemiglutarate-Val-Ala-α-amanitin (**16**)

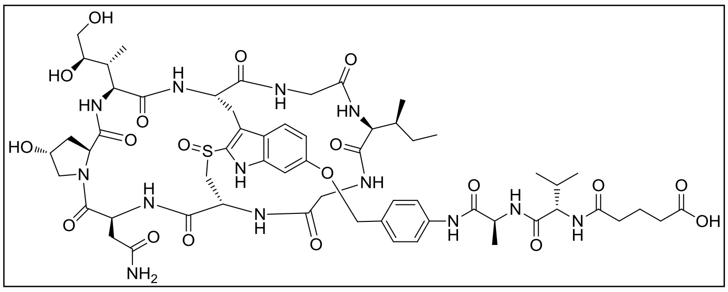

Compound **12b** (5 mg, 3.9 µmol, 1 equiv) was cooled to 0 ºC under a nitrogen atmosphere. Glutaric anhydride (0.65 mg, 5.7 µmol, 1.5 equiv), DMAP (0.12 mg, 0.9 µmol, 0.25 equiv) and iPr_2NEt (1 µL, 5.7 µmol, 1.5 equiv) were added as stock solutions in DMF and the reaction was stirred at 0 ºC for a few minutes. Then the mixture was warmed to room temperature and stirred overnight under a nitrogen atmosphere. The solvent was evaporated under high vacuum and the crude was purified by semipreparative HPLC [Waters Atlantis 21 mm × 10 cm column; gradient: 100% (H_2O + 0.1 % CF_3COOH)/0% (CH_3CN + 0.1% CF_3COOH) to 50% (H_2O + 0.1% CF_3COOH)/50% (CH_3CN + 0.1% CF_3COOH) in 9 minutes; t_R: (product): 8.6 min]. The purified compound was then freeze-dried to afford compound **16** as a white solid (4.92 mg, 98%

yield). MS (MALDI-TOF): *m/z* calcd. for [$C_{59}H_{82}N_{13}O_{19}S$]$^+$: 1309.42 [*M* + H]$^+$; found: 1309 (SA matrix); *m/z* calcd. for [$C_{59}H_{81}N_{13}NaO_{19}S$]$^+$: 1331.40 [*M* + Na]$^+$; found: 1331.7 (HCCA matrix), 1331 (SA matrix); *m/z* calcd. for [$C_{59}H_{81}N_{13}Na_2O_{19}S_2$]$^{2+}$: 1354.39 [*M* + 2 Na]$^{2+}$; found: 1353.7 (HCCA matrix), 1353 (SA matrix).

Cyclo[DKP-RGD]-Val-Ala-α-amanitin (**9**)

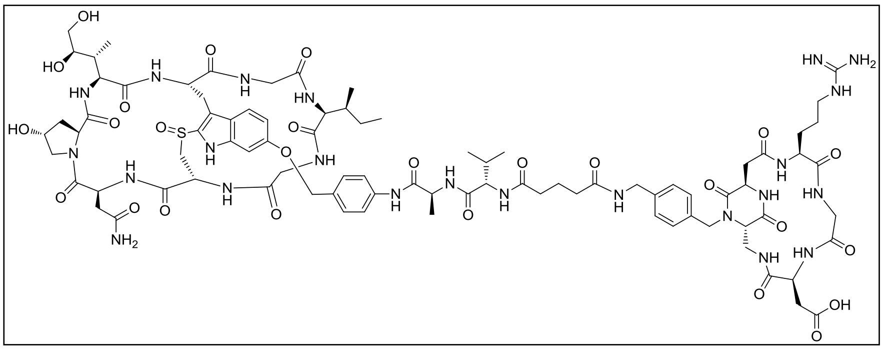

Compound **16** (8.46 mg, 6.5 µmol, 1 equiv) was dissolved in 80 µL of DMF and cooled at 0 ºC under nitrogen conditions. *N*-Hydroxysuccinimide (1.11 mg, 9.7 µmol, 1.5 equiv) and DIC (1.5 µL, 9.7 µmol, 1.5 equiv) were added (the two incorporated reagents were prepared as stock solutions in DMF) and the reaction was stirred at 0 ºC for a few minutes. Then the mixture was warmed to room temperature and stirred overnight under a nitrogen atmosphere. Volatiles were then removed in vacuo to give a solid, which was re-dissolved in PBS (210 µL, pH 7.5) and DMF (30 µL) and cooled to 0 ºC. A solution of NH_2CH_2-cyclo[DKP-RGD] (**2**) (2.78 mg, 3.23 µL, 0.5 equiv) in phosphate buffer (150 µL, pH 7.5) was then added to the previous solution, and the pH was adjusted to 7.3–7.6 by adding small aliquots of aqueous NaOH (0.2 M) during the first hours of reaction, until a stable value was observed. The solution was concentrated, and the crude residue was purified by semipreparative HPLC [Waters Atlantis 21 mm × 10 cm column; flow: 15 mL/min, gradient: 100% (H_2O + 0.1% CF_3COOH)/0% (CH_3CN + 0.1% CF_3COOH) to 50% (H_2O + 0.1% CF_3COOH)/50% (CH_3CN + 0.1% CF_3COOH) in 9 minutes; t_R: (product): 8.3 min]. The purified compound was then freeze-dried to afford compound **9** as a white solid (4.85 mg, 74% of yield).

MS (ESI+) *m/z* calcd. for [$C_{86}H_{119}N_{23}O_{26}S$]$^{2+}$: 960.92 [*M* + 2H]$^{2+}$; found: 960.76; *m/z* calcd. for [$C_{86}H_{118}N_{23}NaO_{26}S$]$^{2+}$: 971.91 [*M* + Na + H]$^{2+}$; found: 972.23; *m/z* calcd. for [$C_{86}H_{117}N_{23}Na_2O_{26}S$]$^{2+}$: 982.9 [*M* + 2Na]$^{2+}$; found: 982.74; HRMS (ESI+): *m/z* calcd. for [$C_{86}H_{118}N_{23}NaO_{26}S$]$^{2+}$: 971.91 [*M* + Na + 1H]$^{2+}$; found: 971.91; *m/z* calcd. for [$C_{86}H_{117}N_{23}Na_2O_{26}S$]$^{2+}$: 982.90 [*M* + 2Na]$^{2+}$; found: 982.90; *m/z* calcd. for

$[C_{86}H_{116}N_{23}Na_3O_{26}S]^{2+}$: 993.89 [*M* + 3Na - H]$^{2+}$; found: 993.89; *m/z* calcd. for $[C_{86}H_{117}N_{23}Na_3O_{26}S]^{3+}$: 662.93 [*M* + 3Na]$^{3+}$; found: 662.93.

Synthesis of cyclo[DKP-isoDGR]-α-amanitin conjugates 8, 10 and 11

Synthesis of cyclo[DKP-isoDGR]-uncleavable-α-amanitin (**8**)

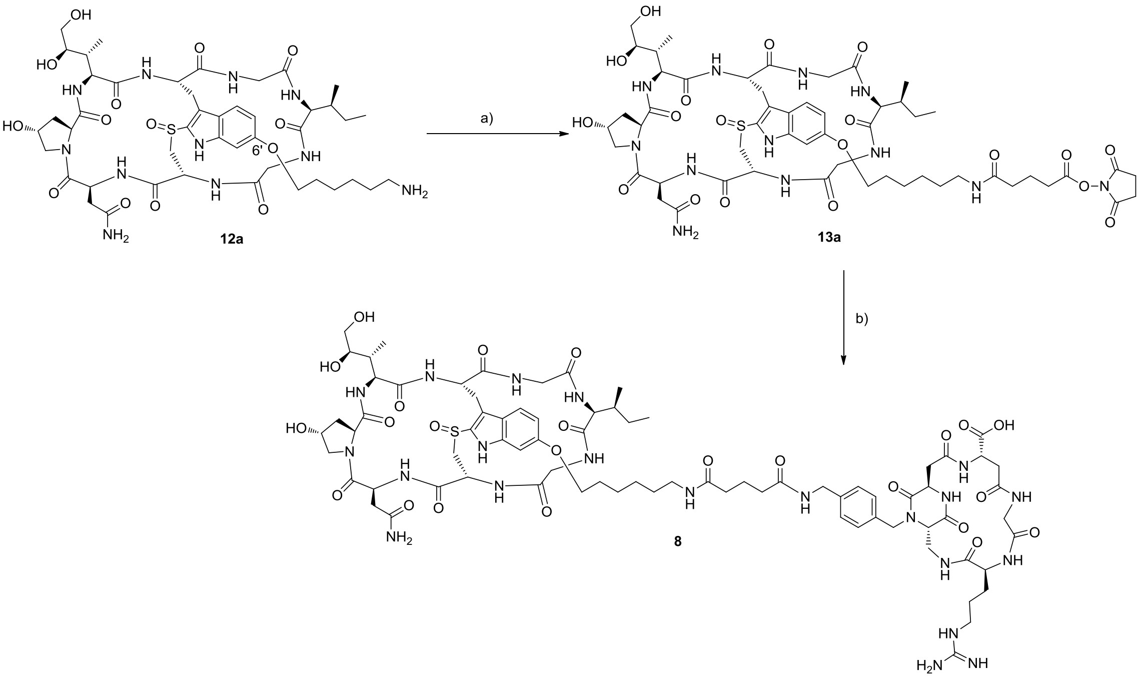

Scheme S3. Synthesis of cyclo[DKP-isoDGR]-uncleavable-α-amanitin (**9**). Reagents and conditions: a) di-*N*-succinimidyl glutarate, iPr_2NEt, DMF, 6 hours, yield: 90%; b) NH_2CH_2-cyclo[DKP-isoDGR] (**4**), PBS (pH 7.5), overnight, yield: 45%.

Glutarate NHS ester-aminohexyl-α-amanitin (**13a**)

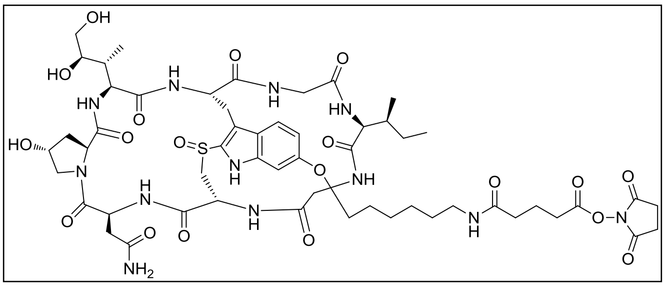

Compound **12a** (11 mg, 10.9 µmol, 1 equiv) was dissolved in 150 µL of dry DMF under nitrogen atmosphere. The solution was cooled to 0 °C, then di-*N*-succinimidyl glutarate (4 mg, 12 µmol. 1.1 equiv) and DIPEA (2 µL, 12 µmol, 1.1 equiv) were added and the mixture was stirred at room temperature for 6 hours. The reaction was monitored by TLC ($CHCl_3$/MeOH/water 65:25:4, cinnamaldehyde staining). The crude was poured into 10 mL precooled MTBE placed in a 10 mL centrifugal tube (the reaction flask was rinsed with 3 × 50 µL of DMF and each rinsing solution was transferred to the MTBE tube). The tube was sealed, vortexed and placed in ice for 10 min. The tube was spun for 3 min at 4500 RPM in a pre-cooled centrifuge. The supernatant was transferred to a 50 mL flask. The pellet was suspended again into 10 mL of MTBE by vortexing and sonication. The tube was placed in ice for 10 mins and the centrifugation was repeated, then the pellet was dried in vacuo. The combined MTBE phases were concentrated under reduced pressure and checked for remaining product by TLC and HPLC. The dried pellet was used in the next step without further purification (12 mg, 90% yield).

Cyclo[DKP-isoDGR]-uncleavable-α-amanitin (**8**)

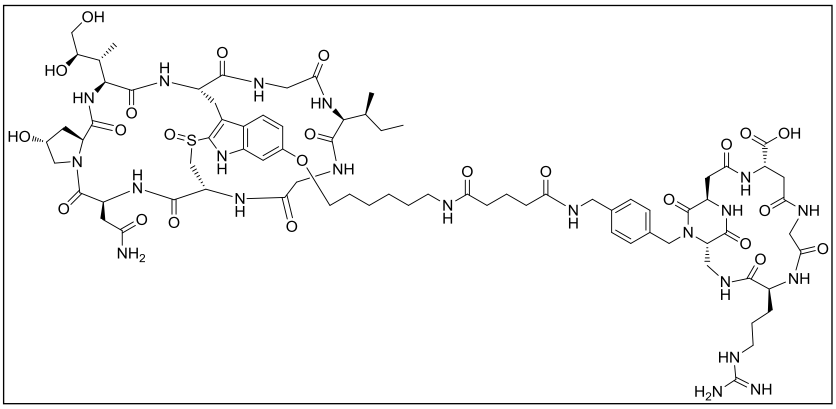

A solution of **4** (5.0 mg, 5.8 µmol, 1 equiv) in 150 µL of PBS was added to a solution of **13a** (12 mg, 10 µmol, 1.7 equiv) in 150 µL of DMF at 0 °C. The pH was adjusted to 7.3–7.6 by adding small aliquots of aqueous NaOH (0.2 M) during the first hours of reaction, until a stable value was observed, and then the reaction mixture was stirred overnight at room temperature.
The solution was directly filtered into a 3 mL vial and purified by preparative HPLC (gradient: from 95% (H_2O + 0.05% CF_3COOH)/5% CH_3CN to 60% (H_2O + 0.05% CF_3COOH)/40% CH_3CN in 14.5 mins), t_R (product): 8.8 min. The purified product was freeze-dried to give the final product as a white solid (4.5 mg, 45% yield). MS (ESI+): *m/z* calcd. for $[C_{77}H_{110}N_{21}O_{24}S]^+$= 1744.77 $[M + H]^+$, found: 1745,67.

Synthesis of *cyclo*[DKP-*iso*DGR]-Val-Ala-α-amanitin (**10**)

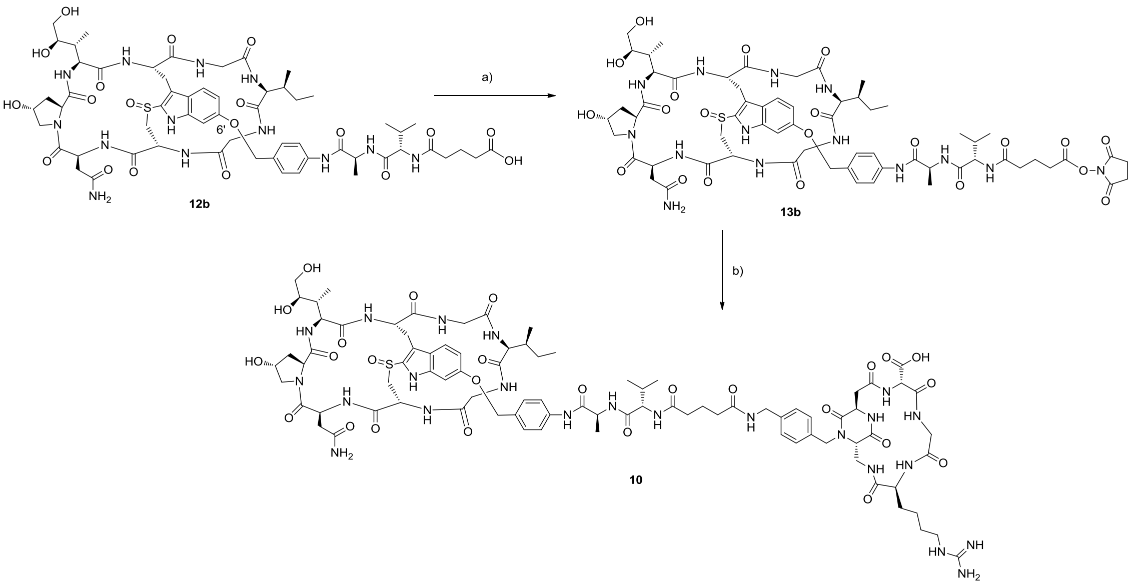

Scheme S4. Synthesis of cyclo[DKP-isoDGR]-Val-Ala-α-amanitin (**10**). Reagents and conditions: a) di-*N*-succinimidyl glutarate, iPr_2NEt, DMF, 6 hours, yield.: 71%; b) NH_2CH_2-cyclo[DKP-isoDGR] (**4**), PBS (pH 7.5), overnight, yield.: 62%.

Glutarate NHS ester-Val-Ala- α-amanitin (**13b**)

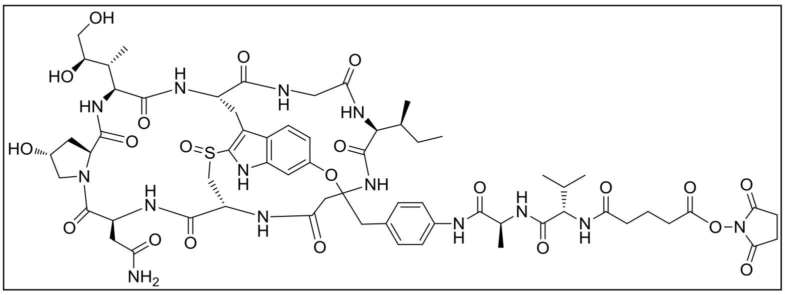

Compound **12b** (10 mg, 8.3 µmol, 1 equiv) was dissolved in 150 µL of dry DMF under nitrogen atmosphere. The solution was cooled to 0 °C, then di-*N*-succinimidyl glutarate (3.2 mg, 9.9 µmol, 1.2 equiv) and DIPEA (1.6 µL, 9.1 µmol, 1.1 equiv) were added and the mixture was stirred at room temperature for 6 hours. The reaction was monitored by TLC ($CHCl_3$/MeOH/water 65:25:4, cinnamaldehyde staining). The crude was poured into 10 mL pre-cooled MTBE placed in a 10 mL centrifugal tube (the reaction flask was rinsed with 3 × 50 µL of DMF and each rinsing solution was transferred to the MTBE tube). The tube was sealed, vortexed and placed in ice for 10 min. The tube was spun for 3 min at 4500 RPM in a precooled centrifuge. The supernatant was transferred to a 50 mL flask. The pellet was suspended again into 10 mL of MTBE by vortexing and sonication. The tube was placed on ice for 10 mins and the centrifugation was repeated. Then the pellet was dried in vacuo. The combined MTBE

phases were concentrated under reduced pressure and checked for remaining product by TLC and HPLC. The dried pellet was used in the next step without further purification (8.3 mg, 71% yield).

Cyclo[DKP-isoDGR]-Val-Ala-α-amanitin (**10**)

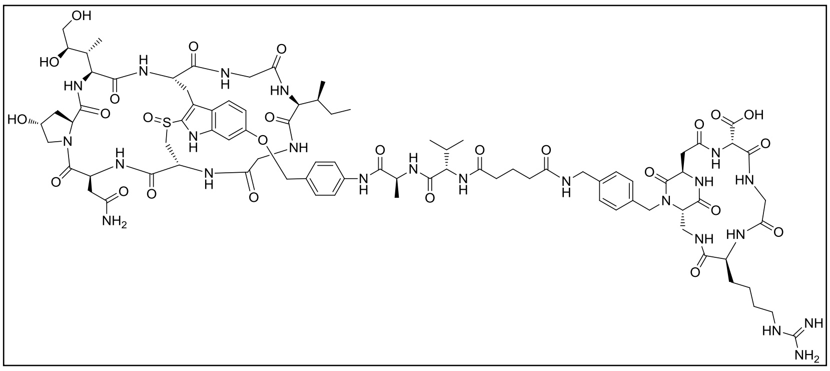

A solution of **4** (3.4 mg, 3.9 µmol, 1 equiv) in 150 µL of PBS was added to a solution of **13b** (8.3 mg, 5.9 µmol, 1.5 equiv) in 150 µL of DMF at 0 °C. The pH was adjusted to 7.3–7.6 by adding small aliquots of aqueous NaOH (0.2 M) during the first hours of reaction (until a stable value was observed), then the reaction mixture was stirred overnight at room temperature. The solution was directly filtered into a 3 mL vial and purified by preparative HPLC (gradient: from 95% (H_2O + 0.05 % CF_3COOH)/5% CH_3CN to 60% (H_2O + 0.05% CF_3COOH)/40% CH_3CN in 14.5 min), t_R (product): 9.2 min. The purified product was freeze-dried to give the final product as a white solid (4.8 mg, 62% yield). MS (ESI+): *m/z* calculated for $[C_{86}H_{118}N_{23}O_{26}S]^+$= 1921.05 $[M + H]^+$, found: 1921.75.

Synthesis of cyclo[DKP-isoDGR]-PEG-4-Val-Ala-α-amanitin (**11**)

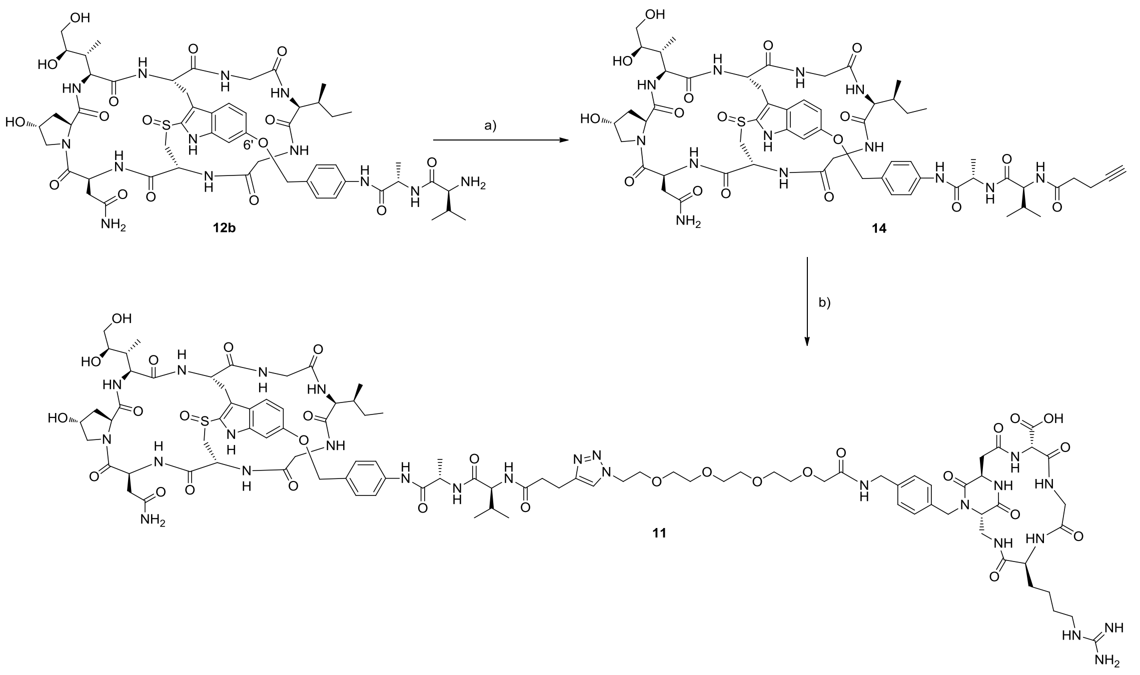

Scheme S5. Synthesis of cyclo[DKP-isoDGR]-PEG-4-Val-Ala-α-amanitin (**11**). Reagents and conditions: a) 4-pentynoic acid NHS ester (**17**), iPr_2NEt, DMF/DCM, overnight, yield.: 88 %; b) N_3-PEG-4-cyclo[DKP-isoDGR] (**18**), DMF/water 1/1, sodium ascorbate, $CuSO_4 \cdot 7H_2O$, overnight, yield: 62%.

4-Pentynoic acid NHS ester (**17**)

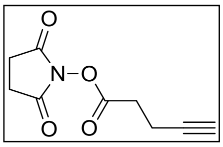

4-Pentynoic acid (10 mg, 0.100 mmol, 1 equiv) was dissolved in 2 mL of dry DCM under argon and cooled to 0 °C. EDC·HCl (23 mg, 0.120 mmol, 1.2 equiv) and NHS (14 mg, 0.120 mmol, 1.2 equiv) were sequentially added to the solution and the reaction mixture was stirred overnight at room temperature. The mixture was diluted in 20 mL of DCM and the organic phase was washed with water (3 × 7 mL), dried over $MgSO_4$, filtrated and concentrated under reduced pressure to give the product as a yellowish oil that was used in next step without further modifications.

4-Pentynoic acid-Val-Ala-α-amanitin (**14**)

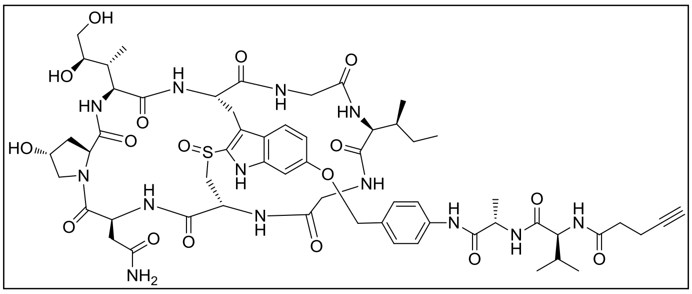

Under argon, DIPEA (1 µL, 6.3 µmol, 1.5 equiv) and compound **17** dissolved in DCM (200 µL) were added to a solution of compound **12b** (5.0 mg, 4.2 µmol, 1 equiv) in 200 µL of DMF kept at 0 °C. The reaction mixture was stirred overnight at room temperature. Volatiles were evaporated under reduced pressure and the crude was dissolved in 500 µL of MeOH and then purified by preparative HPLC (gradient: from 95% (H_2O + 0.05% CF_3COOH) / 5% (CH_3CN) to 0% (H_2O + 0.05% CF_3COOH)/100% (CH_3CN) in 15 mins), t_R product = 7.97 min. The collected fraction was concentrated under reduced pressure and freeze-dried from water/ACN 1/1 to afford the product as a white solid (4.7 mg, 88% yield). MS (ESI+): *m/z* calculated for $[C_{59}H_{80}N_{13}O_{17}S]^+$= 1273.54 $[M + H]^+$, found: 1274.55.

14-Azido-3,6,9,12-tetraoxatetradecanoic acid NHS ester (**19**)

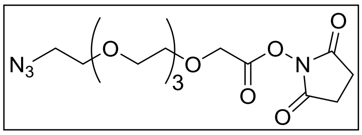

100 µL (0.05 mmol, 1 equiv) of a 0.5 M solution of 14-azido-3,6,9,12-tetraoxatetradecanoic acid (~0.5 M in *tert*-butyl methyl ether) were diluted in 100 µL of dry DCM under argon and cooled to 0 °C. EDC·HCl (12,5 mg, 0.065 mmol, 1.3 equiv) and NHS (7.5 mg, 0.065 mmol, 1.3 equiv) were sequentially added to the solution and the reaction mixture was stirred overnight at room temperature. The mixture was diluted in 10 mL of DCM and the organic phase was washed with water (3 × 3 mL), dried over $MgSO_4$, filtrated and concentrated under reduced pressure to give the product as a transparent oil (18.2 mg, 97% yield). The crude product was used in the next step without further modifications.

N_3-PEG-4-cyclo[DKP- isoDGR] (**18**)

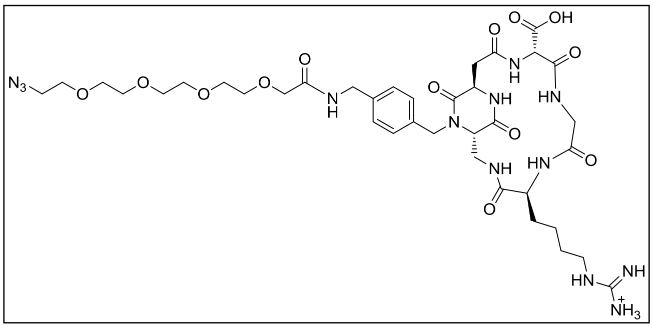

To a solution of compound **19** (9.2 mg, 24 µmol, 2 equiv) in 500 µL of ACN was added H_2NCH_2-*cyclo*[DKP-*iso*DGR] (**4**, 10.5 mg, 12 µmol, 1 equiv) dissolved in 500 µL of PBS (pH 7) was added. The pH was adjusted to 7.4 with a solution of NaOH (0.2 M) and the reaction was stirred overnight at room temperature. The mixture was filtered into a 3 mL vial, centrifuged and purified by preparative HPLC (gradient: from 95% (H_2O + 0.05% CF_3COOH)/5% CH_3CN) to 60% (H_2O + 0.05% CF_3COOH)/40% CH_3CN in 14.5 min, t_R product = 8.3 min). The collected fraction was concentrated under reduced pressure and freeze-dried from 1:1 water/CH_3CN to afford the product as a white solid (5.5 mg, 46% yield). MS (ESI+): *m/z* calculated for $[C_{37}H_{56}N_{13}O_{13}]^+$= 890.41 $[M + H]^+$, found: 890.50.

Cyclo[DKP-isoDGR]-PEG-4-Val-Ala- α-amanitin (**11**)

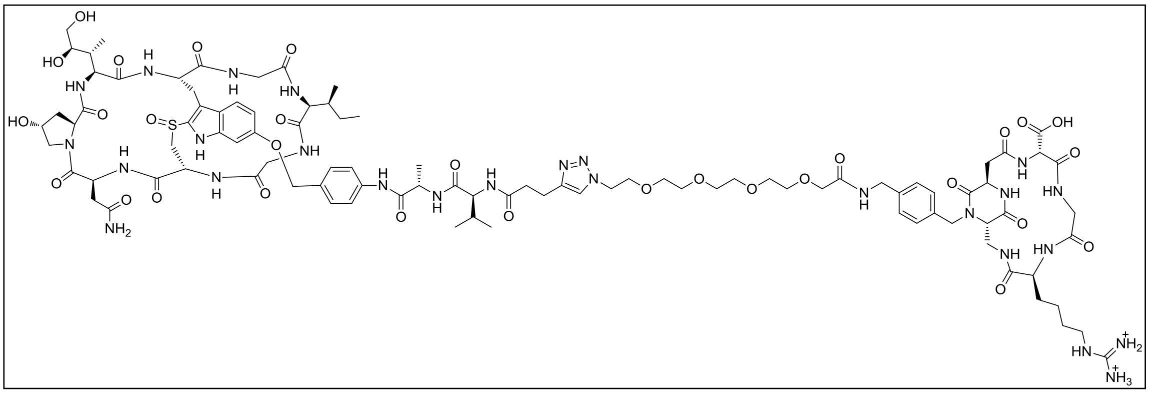

To a solution of **14** (2.9 mg, 2.3 µmol) in 1 mL of DMF at 0 °C, compound **16** (2.7 mg, 2,7 µmol) dissolved in 1 mL of water, sodium ascorbate (0.2 mg, 0.88 µmol) and $CuSO_4·7\ H_2O$ (0.25 mg, 0.44 µmol) were sequentially added. The solution, which turned light yellow, was stirred overnight at room temperature until the color changed to light blue.

Volatiles were evaporated under reduced pressure and the crude was re-dissolved in 400 µL of MeOH, filtered and purified by preparative HPLC (gradient: from 95% (H_2O + 0.05% CF_3COOH)/5% (CH_3CN + 0.05% CF_3COOH) to 0% (H_2O + 0.05% CF_3COOH)/100% (CH_3CN + 0.05% CF_3COOH) in 15 min, t_R product = 7.2 min). The collected fraction was concentrated under reduced pressure and freeze-dried from 1:1 water/CH_3CN to afford the product as a white solid (3.33 mg, 62% yield). MS (ESI+): *m/z* calculated for $[C_{96}H_{136}N_{26}O_{30}S_2]^{2+}$= 1082.48 $[M + 2H]^{2+}$, found: 1082.92.

HPLC traces of the final compounds

Cyclo[DKP-RGD]-uncleavable-α-amanitin (**7**)

Waters Atlantis 21 mm × 10 cm column; gradient: 100% (H_2O + 0.1% CF_3COOH)/0% (CH_3CN + 0.1% CF_3COOH) to 50% (H_2O + 0.1% CF_3COOH)/ 50% (CH_3CN + 0.1% CF_3COOH) in 9 minutes; t_R: (product): 8 min.

Purity: 95%.

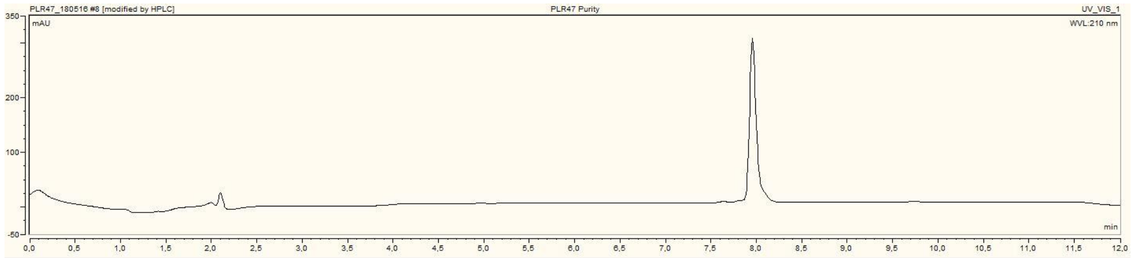

Cyclo[DKP-isoDGR]-uncleavable-α-amanitin (**8**)

Phenomenex Luna C-18(2) column 10 µm, 250 × 21.2 mm, with precolumn at 30 mL/min flow rate; gradient: 95% (H_2O + 0.05% CF_3COOH)/5% CH_3CN to 60% (H_2O + 0.05% CF_3COOH)/40% CH_3CN in 14.5 mins, t_R (product): 8.8 min.

Purity: 96.2%

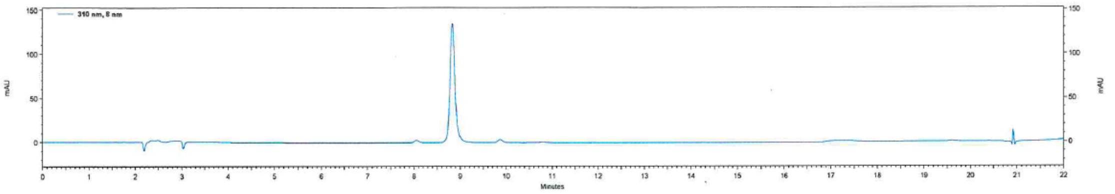

Cyclo[DKP-RGD]-Val-Ala-α-amanitin (**9**)

Waters Atlantis 21 mm × 10 cm column; flow: 15 mL/min, gradient: 100% (H_2O + 0.1% CF_3COOH)/0% (CH_3CN + 0.1% CF_3COOH) to 50% (H_2O + 0.1% CF_3COOH)/50% (CH_3CN + 0.1% CF_3COOH) in 9 minutes; t_R: (product): 8.3 min.

Purity: 99.6%.

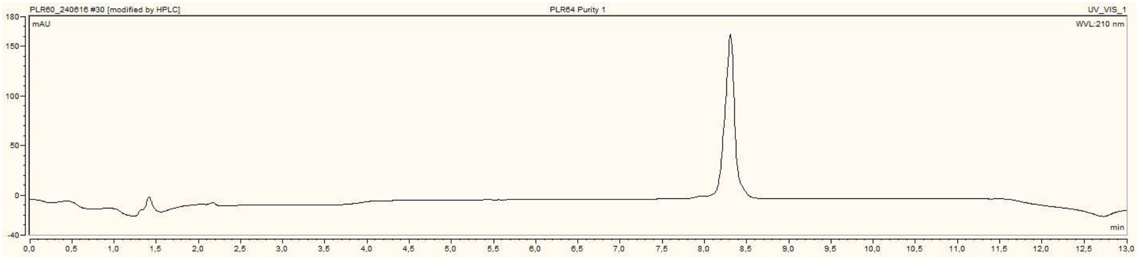

Cyclo[DKP-isoDGR]-Val-Ala-α-amanitin (**10**)

Phenomenex Luna C-18(2) column 10 µm, 250 × 21.2 mm, with precolumn at 30 mL/min flow rate; gradient: 95% (H_2O + 0.05% CF_3COOH)/5% CH_3CN to 60% (H_2O + 0.05% CF_3COOH)/40% CH_3CN in 14.5 mins, t_R (product): 9.2 min.

Purity: 100%.

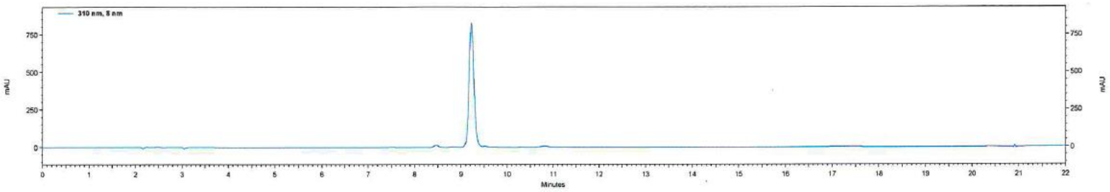

Cyclo[DKP-isoDGR]-PEG-4-Val-Ala- α-amanitin (**11**)

Phenomenex Luna C-18(2) column 10 µm, 250 × 21.2 mm, with precolumn at 30 mL/min flow rate; gradient: 95% (H_2O + 0.05% CF_3COOH)/5% CH_3CN to 100% CH_3CN in 15 mins, t_R (product): 7.2 min

Purity: 89.9%

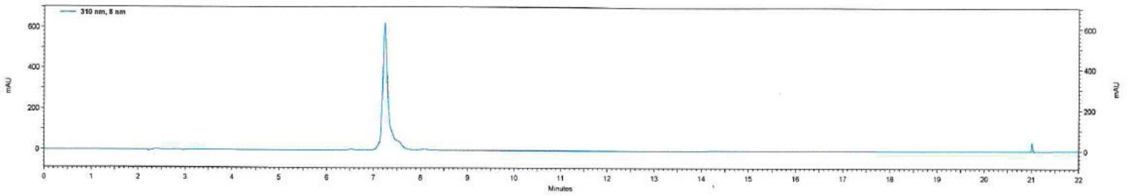

ESI–MS, MALDI–TOF–MS and HRMS Spectra

α-Amanitin-aminohexyl-hemiglutarate (**15**)

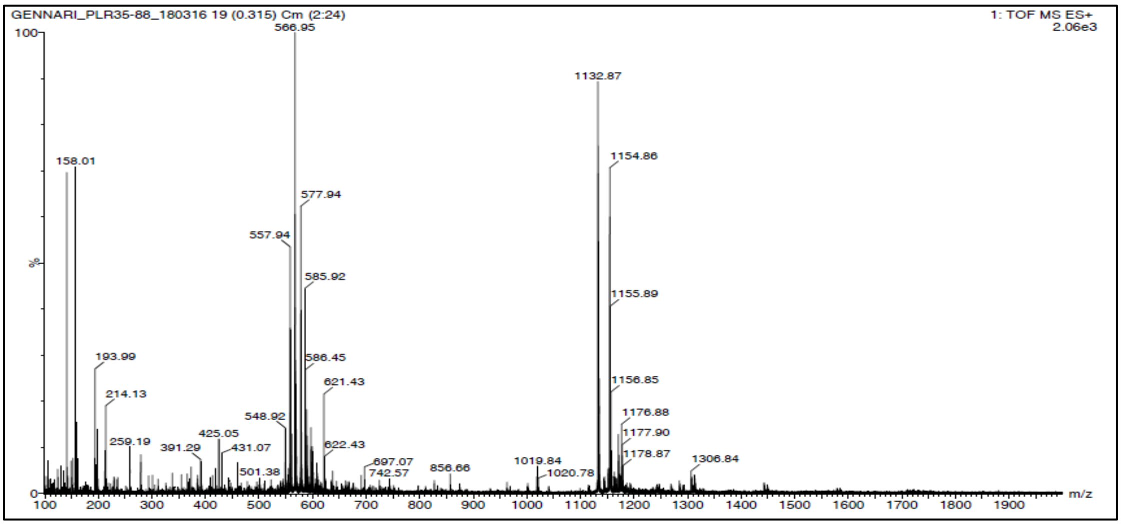

Cyclo[DKP-RGD]-uncleavable-α-amanitin (**7**)

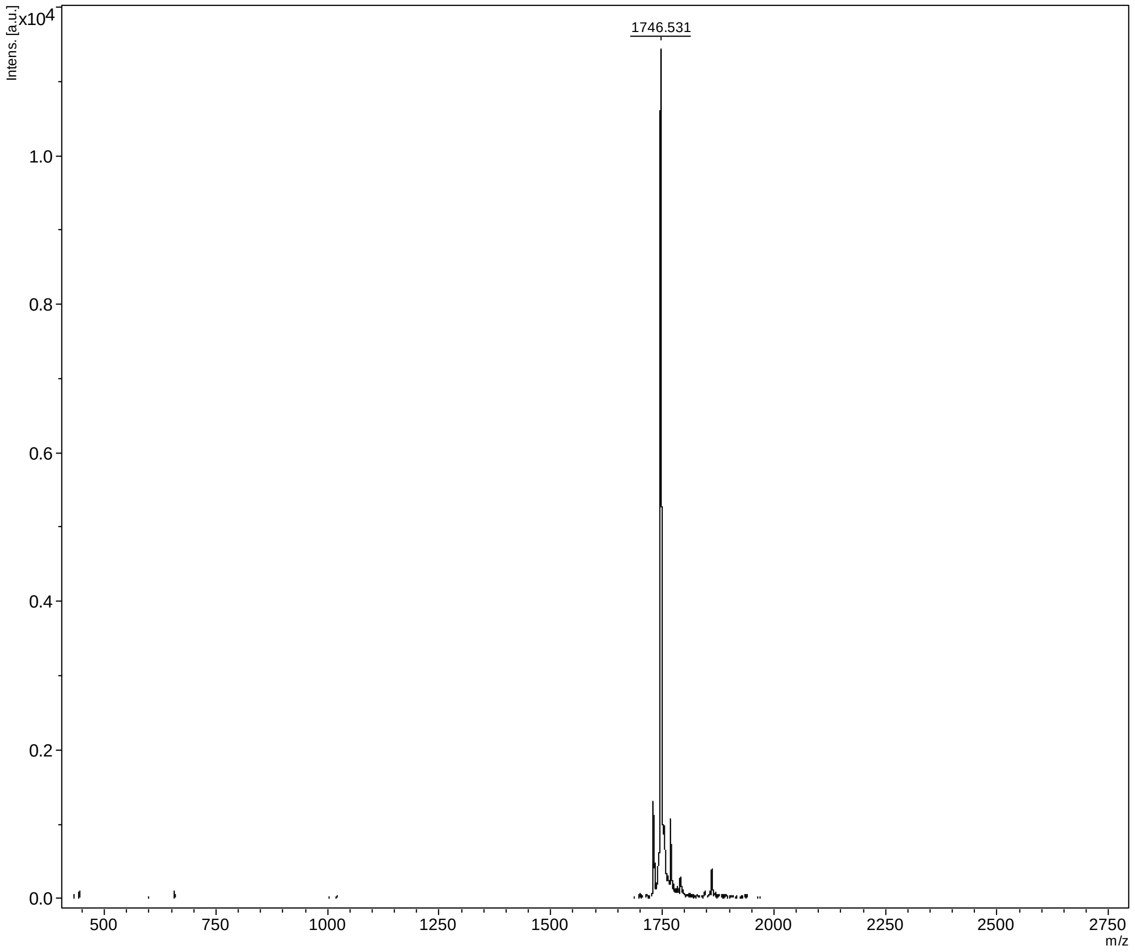

α-Amanitin-aminohexyl-hemiglutarate (**16**)

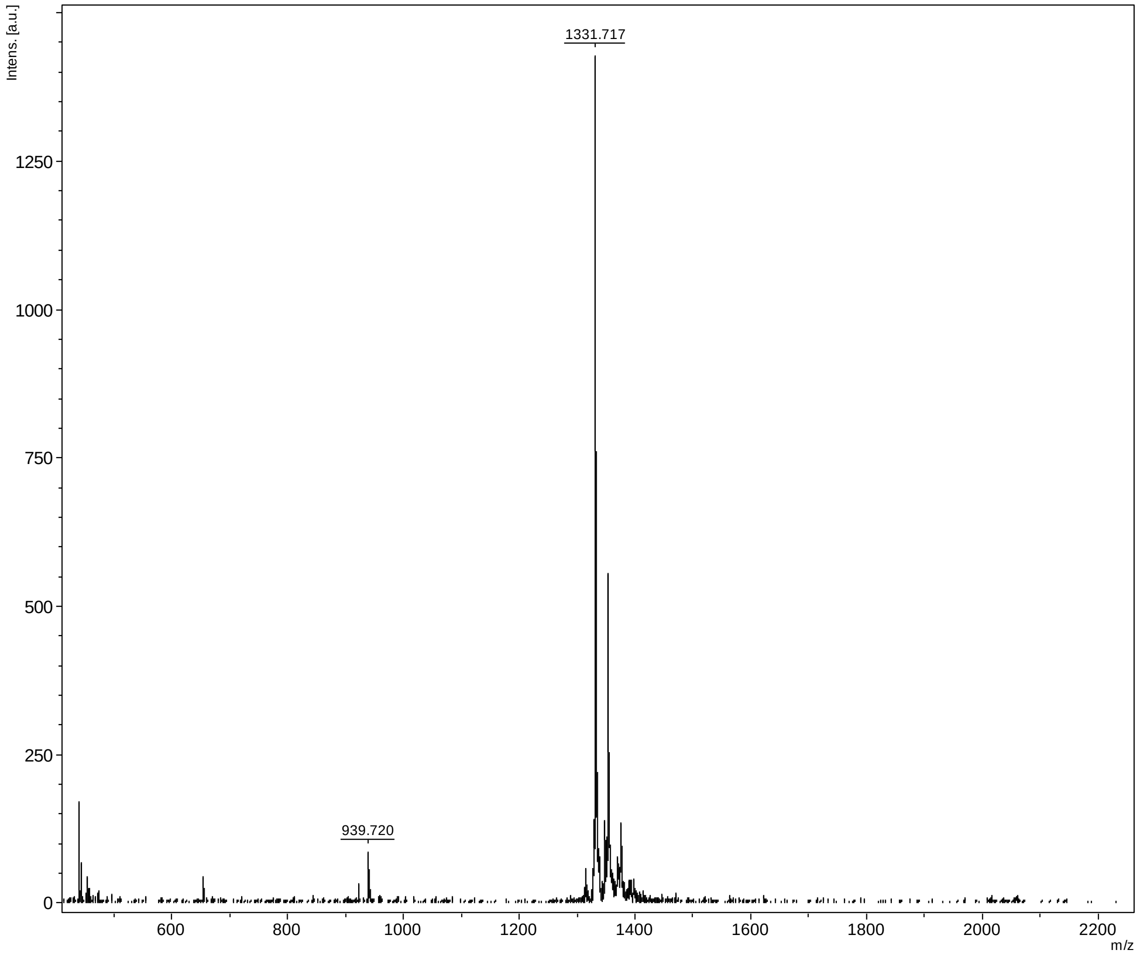

Cyclo[DKP-RGD]-Val-Ala-α-amanitin (**9**)

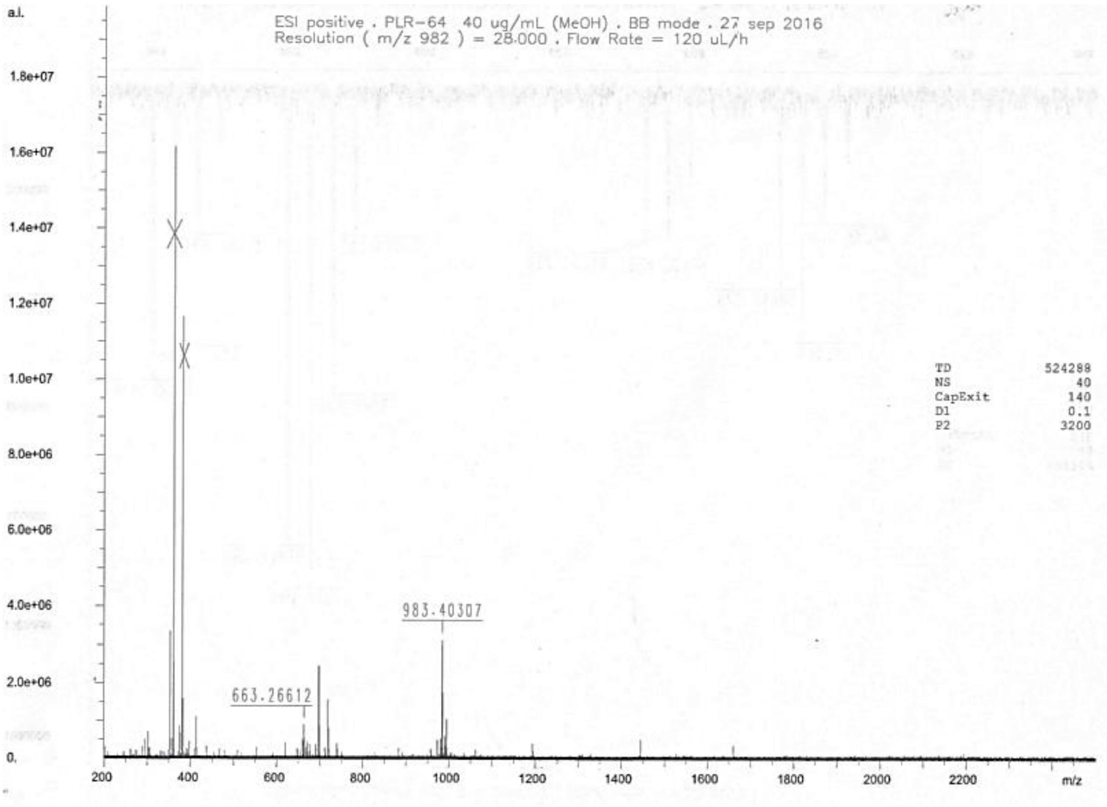

Cyclo[DKP-isoDGR]-uncleavable-α-amanitin (**8**)

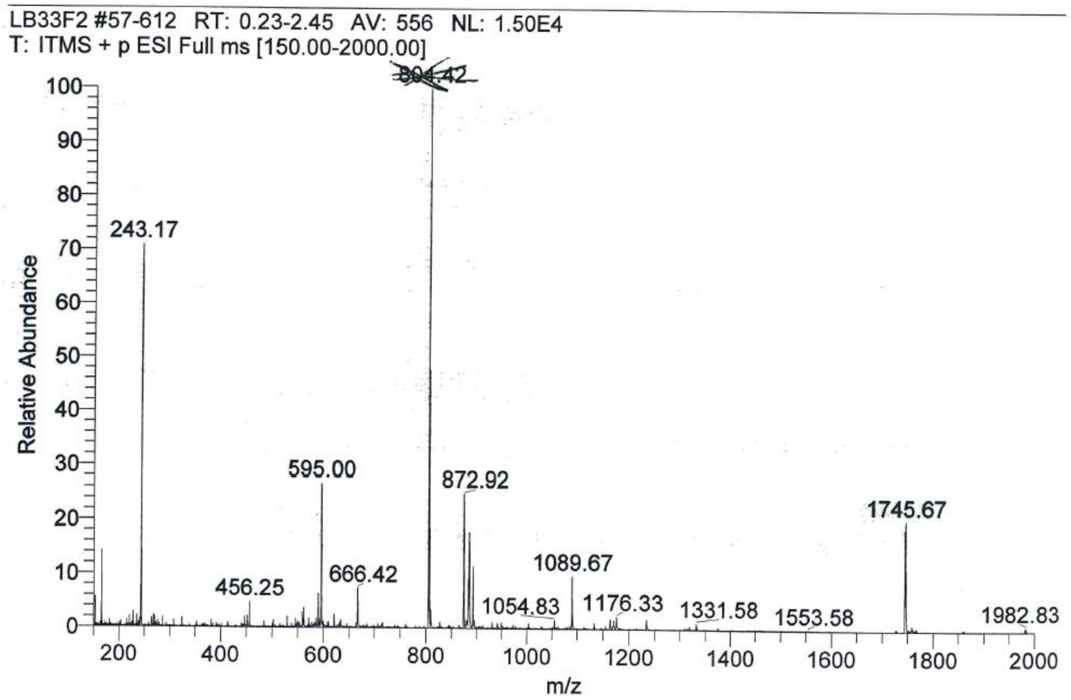

Cyclo[DKP-isoDGR]-Val-Ala-α-amanitin (**10**)

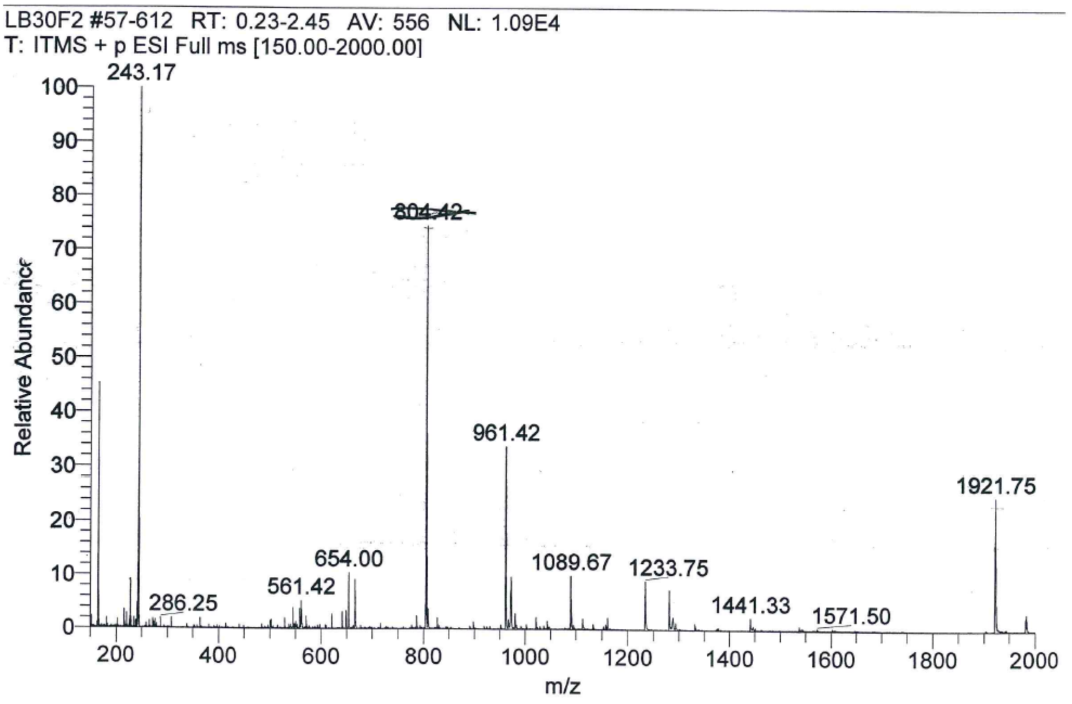

Cyclo[DKP-isoDGR]-PEG-4-Val-Ala- α-amanitin (**11**)

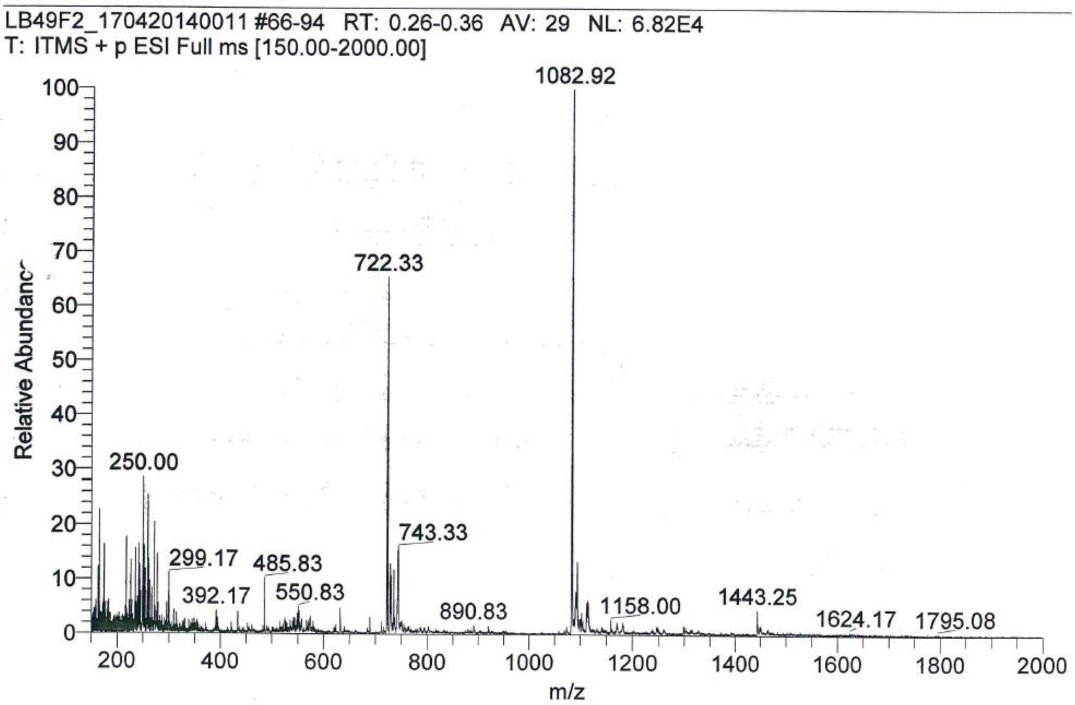

^{1}H NMR and ^{13}C NMR spectra

α-Amanitin-aminohexyl-hemiglutarate (**15**)

^{1}H NMR (400 MHz, D_2O)

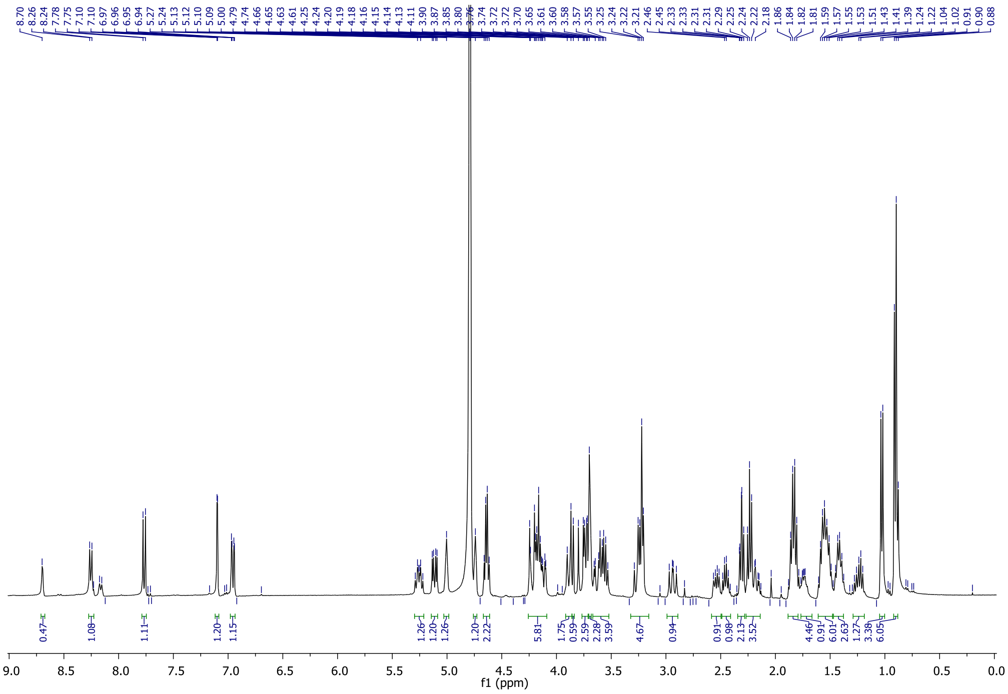

^{13}C NMR (101 MHz, D_2O)

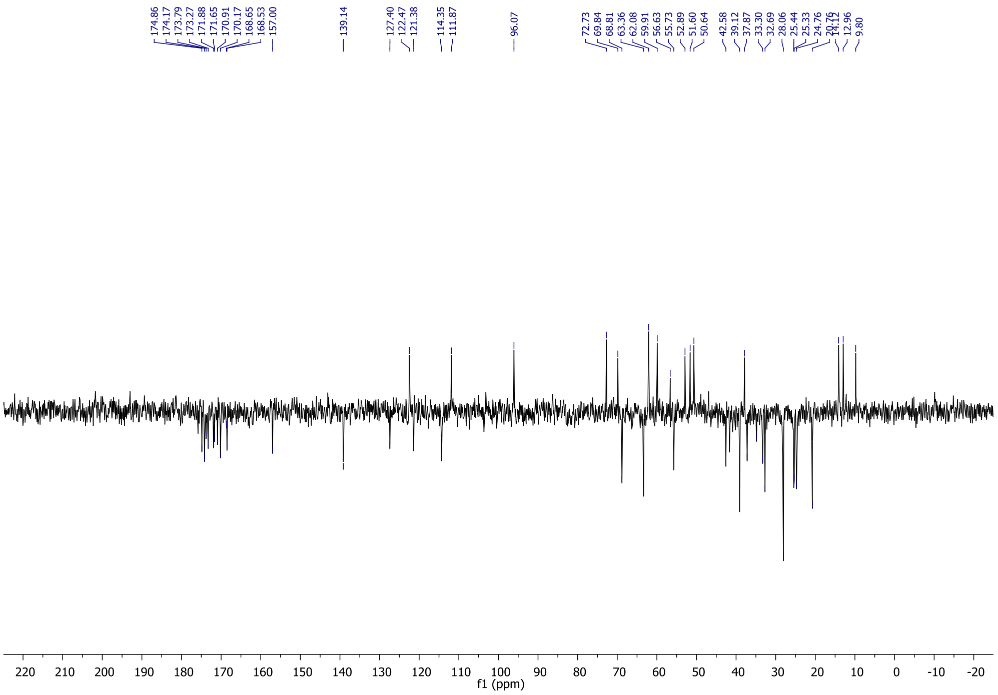

Cyclo[DKP-RGD]-uncleavable-α-amanitin (**7**)

^{1}H NMR (400 MHz, D_2O)

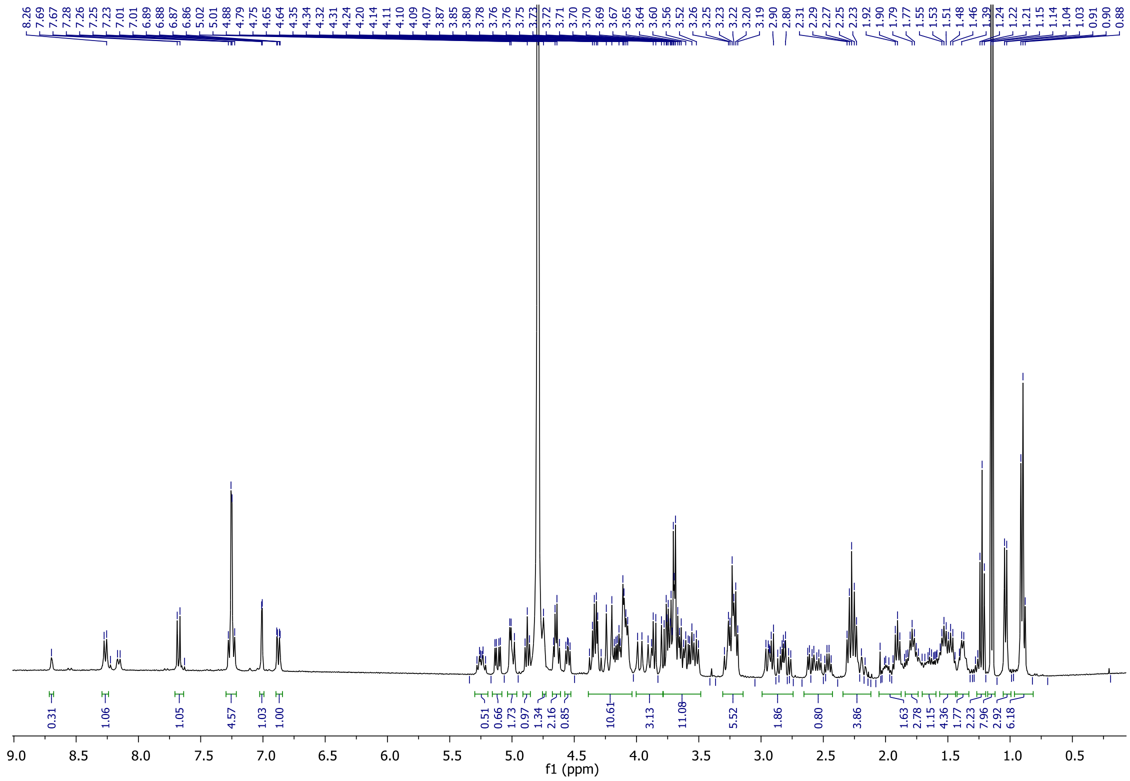

Supplement: File 1 — Experimental details, characterization data and copies of spectra. [file Beilstein_J_Org_Chem-14-407-s001.pdf]
